# The Impact of Directly Observed Therapy on Successful Tuberculosis Treatment: A Statistical Analysis of National Notifications Data

**DOI:** 10.1093/ofid/ofag434

**Published:** 2026-07-16

**Authors:** Ryan Race Thompson, Do Kyung Ryuk, Daniele Maria Pelissari, Luiza Ohana Harada, José Nildo de Barros Silva, Patricia Bartholomay, Fernanda Dockhorn Costa Johansen, Mauro Sanchez, Ted Cohen, Marcia C Castro, Nicolas A Menzies

**Affiliations:** Department of Global Health and Population, Harvard TH Chan School of Public Health, Boston, Massachusetts, USA; Department of Preventive Medicine, Seoul National University College of Medicine, Seoul, Republic of Korea; Health and Environment Surveillance Secretariat, Ministry of Health, Brasília, Brazil; Health and Environment Surveillance Secretariat, Ministry of Health, Brasília, Brazil; Institute of Scientific and Technological Communication and Information in Health, Oswaldo Cruz Foundation, Rio de Janeiro, Brazil; Health and Environment Surveillance Secretariat, Ministry of Health, Brasília, Brazil; Health and Environment Surveillance Secretariat, Ministry of Health, Brasília, Brazil; Núcleo de Medicina Tropical, University of Brasília, Brasília, Brazil; Department of Epidemiology of Microbial Diseases, Yale School of Public Health, New Haven, Connecticut, USA; Department of Global Health and Population, Harvard TH Chan School of Public Health, Boston, Massachusetts, USA; Department of Global Health and Population, Harvard TH Chan School of Public Health, Boston, Massachusetts, USA

**Keywords:** directly observed therapy, disease surveillance data, Latin America, tuberculosis

## Abstract

**Background:**

Directly Observed Therapy (DOT) is a recommended modality for tuberculosis (TB) treatment, yet is underutilized in many settings. Although DOT has been evaluated in clinical trials, less is known about its effectiveness in routine programs. We estimated the contribution of DOT to TB treatment success in Brazil.

**Methods:**

Using data from Brazil's National Disease Notification System for 2015–2018, we fit regression models estimating the relationship between DOT coverage and individual treatment success, leveraging municipality-level variation in DOT usage to avoid confounding due to nonrandom DOT receipt at the individual level. Models were adjusted for demographic and clinical covariates, using generalized estimating equations to account for correlation of outcomes within municipalities. Fitted models were used to project the impact of expanded DOT coverage, with robustness tested using alternative specifications.

**Results:**

The study included 278 007 individuals from 4783 municipalities. Average DOT coverage was 45.3%, with significant geographic variability. Treatment success was 86.9% with DOT and 73.2% without DOT. DOT coverage was associated with a 1.46 (95% confidence interval: 1.38, 1.55) odds ratio (OR) of treatment success. ORs ranged from 1.11 to 2.52 under alternative specifications, consistently indicating a positive impact. Scenario analyses indicated that reducing DOT nonutilization in each municipality by 50% would reduce the proportion of unsuccessful treatment outcomes by 7.1% (6.1, 8.2), equivalent to an average 5.5 (4.7, 6.3) percentage point increase in success probability for individuals newly receiving DOT.

**Conclusions:**

In this routine programmatic setting, higher DOT utilization was associated with better treatment outcomes, underscoring the importance of adherence-supported interventions for TB care.

## Teaser

Using Brazil's national tuberculosis surveillance data, we found directly observed therapy significantly improved treatment success rates. Our findings demonstrate that scale-up of DOT coverage could substantially improve outcomes, supporting strategic policy investment in patient-centered approaches to optimize tuberculosis treatment.

Tuberculosis (TB) is a major public health challenge, with an estimated 10.8 million individuals developing TB in 2023 globally [1]. The recommended treatment for drug-susceptible TB involves a standardized multidrug regimen lasting 6 months or longer, needing high levels of adherence to ensure successful outcomes [2]. However, many individuals do not complete this regimen [3–5].

The current approach to monitoring people undergoing TB treatment, directly observed therapy (DOT), has been promoted by the World Health Organization since the 1990s to improve adherence and reduce loss to follow-up [6]. With DOT, healthcare professionals or trained volunteers observe individuals as they take their medication, confirming adherence and supporting completion of the regimen [6]. Early DOT studies saw substantial improvements in TB outcomes compared to non-DOT approaches. In China, a 1-year DOT pilot study improved treatment success rates by over 40% [6], and a subsequent consensus statement predicted DOT could increase treatment completion to over 90% [7]. However, recent evidence has been mixed, with a 2015 review of 11 clinical trials finding DOT to produce small, nonsignificant improvements in treatment completion and cure rates [8]. DOT requires substantial effort from clinic staff and people receiving treatment, with ongoing debate about whether the benefits of DOT justify the costs.

DOT has been used for TB care in Brazil since the 1990s. However, coverage remains low in many areas, with some municipalities reporting no use of DOT. Advances in DOT, such as remote monitoring through digital adherence technologies like 99DOTS and video call observations [9–11], have reduced barriers to implementing DOT and may allow wider use. A recent study of national TB treatment outcomes found DOT receipt to be the single most important factor in predicting whether someone will successfully complete treatment [12]. However, it's unclear whether this relationship is causal, and what impact efforts to increase DOT coverage would have on TB outcomes.

In this analysis, we used data from Brazil's National Disease Notification System to evaluate the impact of receiving DOT on successful TB treatment and explored how increasing DOT coverage could improve TB treatment outcomes. Prior DOT evaluations relied either on individual-level DOT receipt, which is vulnerable to confounding, or on ecological comparisons of program-level DOT coverage and aggregate outcomes, often in more limited geographic settings. Our study extends this literature by using municipality-level DOT coverage as a contextual exposure linked to individual treatment outcomes across nearly all Brazilian municipalities, while adjusting for detailed individual and municipality-level covariates and quantifying potential gains from specific DOT scale-up scenarios. Understanding these dynamics is valuable for developing effective policies to enhance TB management and reduce disease burden across diverse populations.

## METHODS

### Study Population and Data

We extracted data from Brazil's National Disease Notification System (Sistema de Informação de Agravos de Notificação, SINAN) [13] on individuals diagnosed with TB disease between January 1, 2015 and December 31, 2018. SINAN includes information on all individuals notified with TB in Brazil, including demographic characteristics, co-prevalent health conditions, risk factors and behaviors, and treatment outcomes. We assigned each TB notification in SINAN to a municipality based on the clinic where they received TB treatment and linked the resulting dataset to municipality-level characteristics including socio-demographic and health systems characteristics. The Brazil Ministry of Health (MOH) defines DOT receipt in SINAN as individuals who had at least 3 doses of treatment observed per week by a healthcare professional, administered at health facilities, an individuals' home, or their workplace, throughout treatment [14]. In Brazil, monitoring medication intake via digital platforms is considered valid for DOT [14].

We excluded individuals recorded as having transferred health facilities during treatment, receiving retreatment following initial treatment failure, diagnosed with drug-resistant TB, recorded as having a change of diagnosis or treatment regimen, or with nonpulmonary TB. Municipalities were excluded if ≥50% of people with TB transferred to another municipality during treatment. We used multiple imputations to account for missingness, implemented with multiple imputations via chained equations (MICE, additional details in Supplementary material) [15]. This produced 10 multiply imputed datasets, which were used for all subsequent analyses.

### Outcome Definition

Treatment success was defined as completing treatment without evidence of ongoing TB disease. Unsuccessful treatment was defined as death, loss to follow-up/abandonment, or completing treatment with evidence of ongoing disease.

### Analysis

The aim of this analysis was to estimate the relationship between receipt of DOT and treatment success, controlling for other factors influencing treatment success. In programmatic data, it is possible that DOT receipt is correlated with individual factors that are independent predictors of treatment success, biasing the relationship between DOT receipt and treatment outcomes. Controlling for observed characteristics may not fully resolve this potential confounding. Therefore, for our main analysis we estimated the relationship between DOT receipt and treatment success based on municipality-level variation in DOT coverage, rather than individual DOT receipt. A directed acyclic graph is provided in the Supplement (Supplementary Figure 8.1). For the primary analysis, we used logistic regression to estimate the odds of successful treatment as a function of municipality-level DOT coverage, treating DOT coverage as a contextual (municipality-level) exposure. We fit these models using generalized estimating equations (GEE) to account for potential correlation of outcomes within municipalities. Both univariate and multivariate models were considered, incorporating both individual- and municipality-level covariates. Covariates were excluded if they had a variance inflation factor >10. Odds ratio (OR) and 95% confidence intervals (CIs) were estimated based on the regression coefficients. The DOT coverage variable was parameterized so that resulting ORs represented the implied odds of treatment success for an individual receiving DOT versus non-DOT.

### Scenario Analysis

Using the fitted logistic regression models, we explored 3 scenarios for scaling up DOT coverage.

1. Reducing the number of individuals not receiving DOT by 50% in each municipality.
2. Increasing DOT coverage in each municipality by 20 percentage points, capped at 100% coverage.
3. Increasing DOT coverage to 80% among municipalities with <80% DOT coverage.

For each scenario, we report the revised estimate of national DOT coverage, the number and percent point change in treatment successes, the percent reduction in unsuccessful treatments, and the percentage point change in treatment success probability for individuals newly receiving DOT. All changes to DOT coverage were implemented at the municipality level, and then the probability of treatment success was re-estimated for each individual. We assumed monotonicity: those on DOT were assumed to stay on DOT, and a fraction of those not originally receiving DOT assumed to switch to DOT.

### Sensitivity Analyses

We tested the robustness of results to alternative analytic specifications. First, a secondary analysis used the same GEE model, replacing municipality DOT coverage with individual DOT receipt. This individual-level OR represents the effect of DOT on treatment outcomes as well as the effect of unobserved individual-level factors correlated with both DOT receipt and treatment success.

Second, we used an alternative model specification (multilevel logistic regression model, MLM) to estimate study outcomes. The MLM included municipality and state random effects (26 states plus the federal district) to account for unmodeled factors contributing to treatment success (details in Supplementary materials).

Third, we explored the impact of missingness in DOT coverage and treatment outcomes. In 2 sensitivity analyses, we assumed all individuals with missing DOT information did, and did not, receive DOT. A third missingness sensitivity analysis considered all individuals with missing outcome information as unsuccessful treatments, matching current MOH definitions [16]. A final missingness sensitivity analysis excluded all individuals with missing data.

Fourth, we investigated 2 alternative outcome variables that contribute to treatment success: death on treatment, and lost to follow-up prior to treatment completion. Both outcomes were operationalized as binary outcomes, and evaluated with the same logistic GEE framework used for the primary outcome.

Last, we explored how results differed across sub-groups of the study sample. Three sub-groups were considered: one excluding individuals with recurrent TB; one considering only individuals treated in primary care; and one considering only individuals with a positive sputum smear microscopy (SSM) result at diagnosis. All regressions and scenario/sensitivity analyses were conducted in R v4.4.3 [17].

### Assessment of Spatial Clustering

Exploratory spatial autocorrelation analysis evaluated geographic clustering of 3 municipality-level indicators: municipality-level DOT coverage, percent TB treatment successes, and the percent of individuals with missing information on DOT receipt. Spatial analyses were performed at the municipality level with spatial weights (the strength of the geographic relationship between a pair of municipalities) using first-order Queen Contiguity (all immediate neighboring municipalities are assigned equal weight) [18]. Global Moran's I assessed whether municipalities with similar indicator values are clustered geographically more than expected by chance, known as global spatial autocorrelation [19]. Statistical significance of Global Moran's I was estimated with Monte Carlo permutation testing (99 999 repetitions, α = .05), which repeatedly redistributes the observed values randomly across municipalities to generate a reference distribution of what Moran's I would look like under no spatial clustering, against which the observed statistic is then compared [19]. Local indicators of spatial association (LISA) statistics, which decompose global spatial autocorrelation into location-specific contributions, were assessed using Local Moran's I to identify specific municipalities driving this clustering of indicators [20]. Municipality-level LISA clusters were identified using permutation-based *P*-values (99 999 permutations), with statistical significance determined using a false discovery rate correction at α = .05 to account for repeat testing [21, 22]. Maps of clusters were generated based on LISA significance. Spatial analyses were conducted in GeoDa v.1.22.0.14 [23].

### Ethics Statement

This study used previously collected, anonymized data, and was determined to be nonhuman subjects research by the Harvard T. H. Chan School of Public Health Institutional Review Board.

## RESULTS

**Figure 1.**
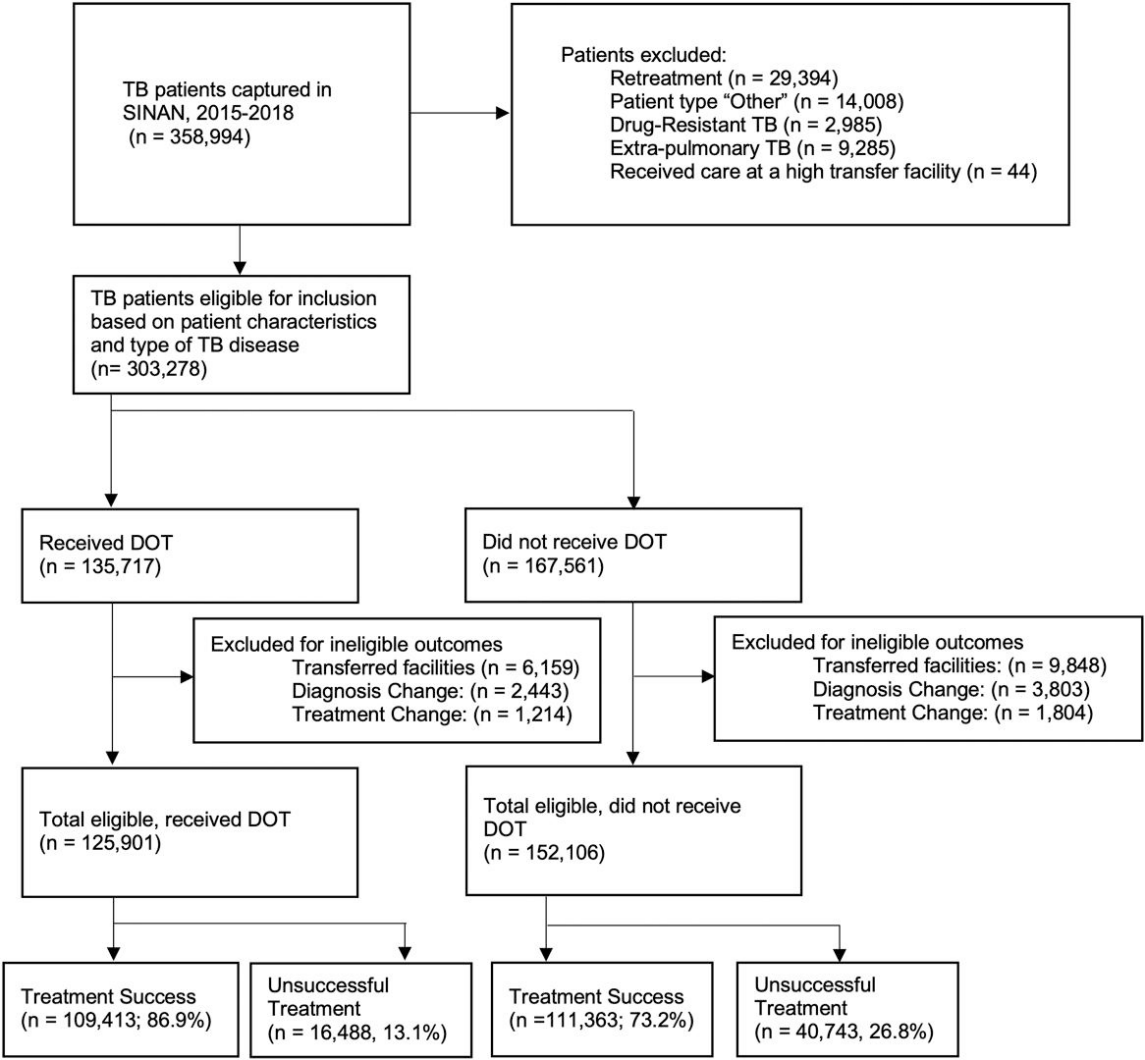
Flow chart of eligibility across study population and by receipt of DOT. *Based on multiple imputation of missing data. Results averaged across the 10 imputations. DOT: Directly Observed Therapy. SINAN, Sistema de Informação de Agravos de Notificação; TB, tuberculosis.

**Figure 2.**
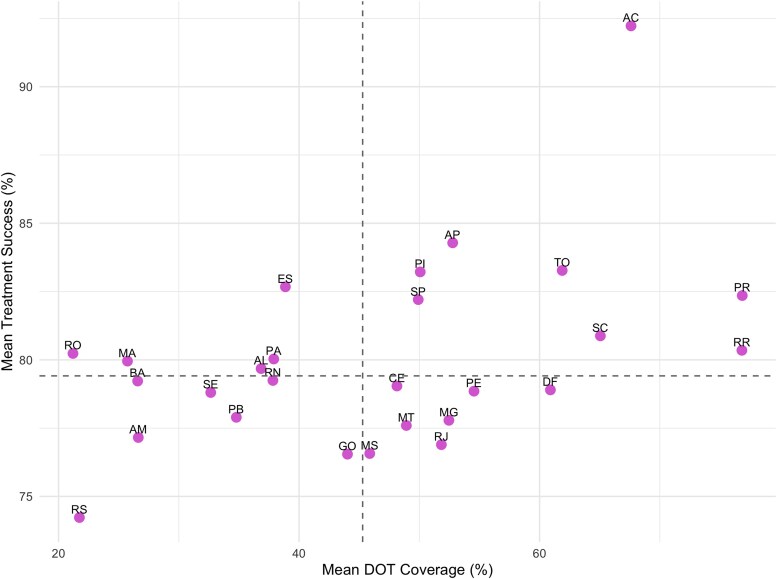
Comparison of mean DOT coverage (the percent of individuals receiving DOT) and mean treatment success (percent of individuals experiencing a successful treatment outcome) by Brazilian state, 2015–2018.* Dotted lines represent the national mean values for DOT coverage (x-axis) and treatment success (y-axis). *Based on multiple imputation of missing data. Results averaged across the 10 imputations. DOT, directly observed therapy. State abbreviations: AC, Acre; AL, Alagoas; AM, Amazonas; AP, Amapá; BA, Bahia; CE, Ceará; DF, Distrito Federal; ES, Espírito Santo; GO, Goiás; MA, Maranhão; MG, Minas Gerais; MS, Mato Grosso do Sul; MT, Mato Grosso; PA, Pará; PB, Paraíba; PE, Pernambuco; PI, Piauí; PR, Paraná; RJ, Rio de Janeiro; RN, Rio Grande do Norte; RO, Rondônia; RR, Roraima; RS, Rio Grande do Sul; SC, Santa Catarina; SE, Sergipe; SP, São Paulo; TO, Tocantins.

**Figure 3.**
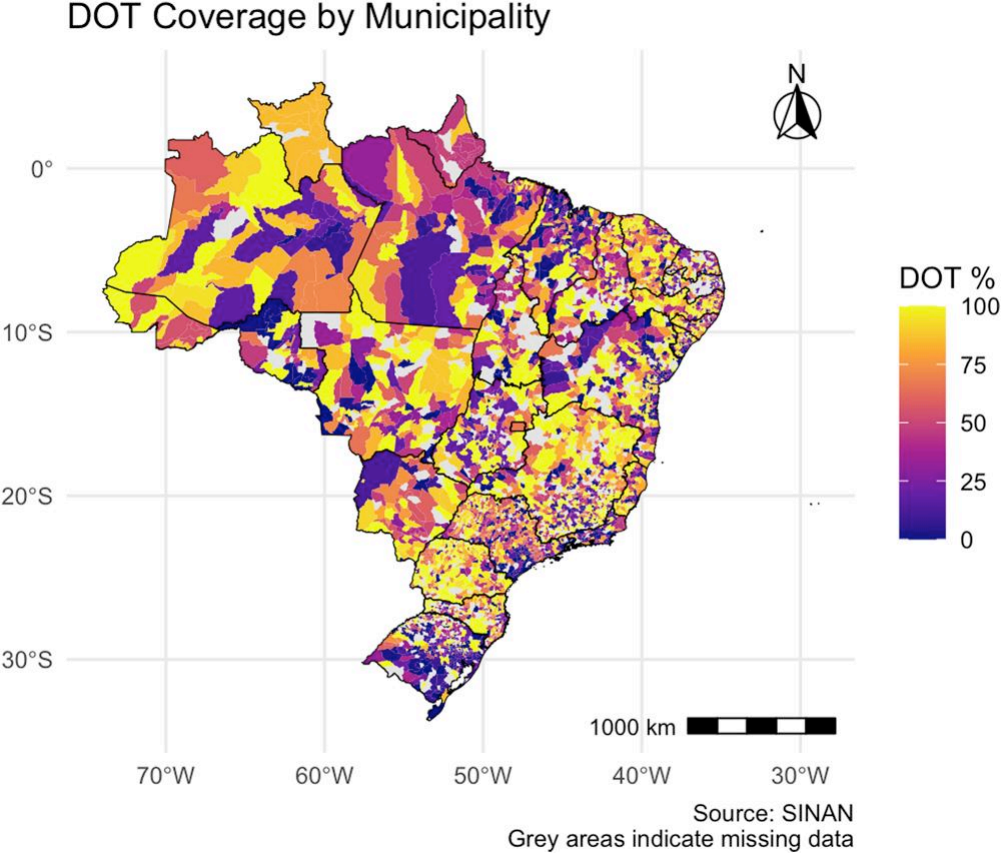
DOT coverage by municipality in Brazil. The map is color coded, with darker (bluer) colors indicating low DOT coverage, and brighter (yellower) colors indicating high DOT coverage. *Based on multiple imputation of missing data. Results averaged across the 10 imputations. DOT: Directly Observed Therapy. SINAN: Sistema de Informação de Agravos de Notificação.

Data for 358 994 individuals with diagnosed TB were included in SINAN from 2015 to 2018, of whom 278 007 were eligible for inclusion, representing 4783 municipalities and all 27 states (Figure 1). Demographic and clinical characteristics of the study sample are presented in Table 1, municipal-level covariates are presented by DOT coverage tercile in the Supplement. There were 220 776 estimated treatment successes (79.4%). DOT receipt was reported for 125 901 individuals (45.3%). There was substantial variation in DOT coverage and treatment success rates at the state and municipality level (Figures 2 and 3), with an interquartile range of 11.1–82.4% for DOT coverage and 53.3–88.2% for treatment success rates across municipalities. Across all individuals in the study cohort, the treatment success rate was 86.9% (109 413/125 901) for DOT recipients and 73.2% (111 363/152 106) for non-DOT recipients (crude OR: 2.43).

**Table 1.** **Demographic and Clinical Characteristics of the Study Sample, Stratified by DOT Receipt** ^a^

	Received DOT (n = 125 901)	Did Not Receive DOT (n = 152, 106)		Received DOT	Did Not Receive DOT
Age category			Culture result		
0–4	1398 (1.1)	1733 (1.1)	Positive	30 753 (24.4)	26 585 (17.5)
5–14	2237 (1.8)	2599 (1.7)	Negative	12 400 (9.8)	12 645 (8.3)
15–24	24 354 (19.3)	26 696 (17.6)	In-Progress	3656 (2.9)	6237 (4.1)
25–44	52 350 (41.6)	62 575 (41.1)	Not performed	78 586 (62.4)	105 931 (69.6)
45–64	33 932 (27.0)	42 961 (28.2)	Missing	506 (0.4)	708 (0.5)
65–74	7441 (5.9)	9787 (6.4)	Chest X-ray		
75–84	3305 (2.6)	4524 (3.0)	Suspected TB	70 134 (55.7)	88 973 (58.5)
85+	884 (0.7)	1231 (0.8)	Normal	7541 (6.0)	10 204 (6.7)
Year of diagnosis			Not performed	48 226 (38.3)	52 929 (34.8)
2015	31 031 (24.6)	36 512 (24.0)	HIV		
2016	31 082 (24.7)	36 755 (24.2)	Positive	8342 (6.6)	15 551 (10.2)
2017	32 646 (25.9)	38 209 (25.1)	Negative	97 744 (77.6)	103 655 (68.1)
2018	31 142 (24.7)	40 630 (26.7)	Other	19 815 (15.7)	32 900 (21.6)
Sex			Alcohol use disorder		
Male	88 635 (70.4)	103 021 (67.7)	Positive	21 634 (17.2)	24 691 (16.2)
Female	37 266 (29.6)	49 085 (32.3)	Negative	98 114 (77.9)	119 655 (78.7)
Race			Other	6153 (4.9)	7760 (5.1)
White	39 546 (31.4)	46 548 (30.6)	Diabetes		
Black	15 644 (12.4)	18 432 (12.1)	Positive	9451 (7.5)	12 153 (8.0)
Asian	966 (0.8)	1064 (0.7)	Negative	110 072 (87.4)	131 710 (86.6)
Mixed	59 018 (46.9)	72 437 (47.6)	Other	6378 (5.1)	8243 (5.4)
Indigenous	2148 (1.7)	941 (0.6)	Prison population		
Other	8579 (6.8)	12 684 (8.3)	Yes	18 015 (14.3)	12 284 (8.1)
Education			No	103 896 (82.5)	133 983 (88.1)
No education	7036 (5.6)	5626 (3.7)	Other	3990 (3.2)	5839 (3.8)
Incomplete one–fourth grade	15 915 (12.6)	16 350 (10.7)	Homelessness		
Complete one–fourth grade	30 857 (24.5)	35 035 (23.0)	Yes	3076 (2.5)	3624 (2.4)
Complete fifth–eighth grade	24 052 (19.1)	26 104 (17.2)	No	118 402 (94.0)	142 402 (93.6)
Complete high school	9480 (7.5)	15 315 (10.1)	Other	4423 (3.5)	6080 (4.0)
Higher education	6454 (5.1)	10 268 (6.8)	Immigrant status		
Other	32 107 (25.5)	43 408 (28.5)	Yes	1640 (1.3)	1506 (1.0)
Form of TB			No	117 808 (93.6)	142 694 (93.8)
New TB case	115 320 (91.6)	139 708 (91.8)	Other	6453 (5.1)	7906 (5.2)
Recurrent TB	10 581 (8.4)	12 398 (8.2)	Smoking		
Health facility level			Yes	27 932 (22.2)	31 494 (20.7)
Primary	88 376 (70.2)	85 593 (56.3)	No	91 255 (72.5)	112 009 (73.6)
Secondary	22 437 (17.8)	47 764 (31.4)	Other	6714 (5.3)	8603 (5.7)
Tertiary	11 773 (9.4)	12 231 (8.0)	Drug use status		
Other	3315 (2.6)	6518 (4.3)	Yes	16 089 (12.8)	17 765 (11.7)
Sputum smear microscopy result			No	102 525 (81.4)	124 955 (82.1)
Positive	68 432 (54.4)	76 210 (50.1)	Other	7287 (5.8)	9386 (6.2)
Negative	23 259 (18.5)	29 851 (19.6)	Treatment outcome		
Not performed	30 929 (24.6)	40 132 (26.4)	Successful	109 413 (86.9)	111 363 (73.2)
Not applicable	3281 (2.6)	5913 (3.9)	Unsuccessful	16 488 (13.1)	40 743 (26.8)

Abbreviations: DOT, directly observed therapy; HIV, human immunodeficiency virus; TB, tuberculosis.

^a^Based on multiple imputation of missing data. Results averaged across the 10 imputations.

### Association Between DOT Coverage and Treatment Success

Table 2 reports OR for the relationship between treatment success rates and municipality-level DOT coverage under several regression models. Under the main analysis model (controlling for all individual and municipality covariates) the odds of treatment success was estimated as 1.46 (95% CI: 1.38, 1.55) for DOT compared to non-DOT. OR were higher under models that controlled for smaller sets of covariates.

**Table 2.** Odds Ratio for Treatment Success With Increased DOT Coverage via Generalized Estimating Equations, and Results of Scenario Analysis for 50% Reduction in DOT Nonreceipt

Outcome	Univariate	Demographic Covariates Only	Selected Baseline Covariates	All Individual Covariates (Including Demographic Covariates)	All Municipality Covariates	All Individual And Municipality Covariates^a^
Odds ratio of treatment success for DOT versus non-DOT	1.76 (1.66, 1.86)	1.70 (1.60, 1.80)	1.52 (1.44, 1.61)	1.50 (1.42, 1.59)	1.69 (1.59, 1.79)	1.46 (1.38, 1.55)
Scenario analysis: 50% reduction in DOT nonreceipt in each municipality (Scenario 1)^b^						
Total increase in treatment successes	6458 (5721, 7246)	6018 (5369, 6705)	4672 (4054, 5282)	4374 (3761, 4976)	6556 (5840, 7271)	4199 (3618, 4823)
Percentage point^c^ increase in treatment success (total program)	2.3 (2.1, 2.6)	2.2 (1.9, 2.4)	1.7 (1.5, 1.9)	1.6 (1.4, 1.8)	2.4 (2.1, 2.6)	1.5 (1.3, 1.7)
Percentage reduction in unsuccessful treatment (total program)	13.7% (12.3, 15.2)	10.7% (9.6, 11.9)	8.4% (7.2, 9.4)	7.8% (6.7, 8.9)	11.0% (9.9, 12.2)	7.1% (6.1, 8.2)
Percentage point^c^ increase in treatment success, (individuals newly receiving DOT)	8.5 (7.5, 9.5)	7.9 (7.0, 8.8)	6.1 (5.3, 6.9)	5.7 (4.9, 6.5)	8.6 (7.7, 9.5)	5.5 (4.7, 6.3)
Percentage reduction in unsuccessful treatments, (individuals newly receiving DOT)	49.8% (44.6, 55.4)	39.0% (34.8, 43.4)	30.5% (26.4, 34.4)	28.4% (24.3, 32.3)	40.2% (35.9, 44.5)	26.0% (22.2, 29.8)

Variables included:

Primary exposure (all models): Municipality-level DOT coverage.

Demographic: age group at TB diagnosis (years); year of TB diagnosis; sex; race; education level.

Individual select baseline covariates: HIV status; prison population; health facility level for treatment.

Individual: TB case type; sputum smear microscopy result; culture result; chest X-ray result; diagnosed with alcohol use disorder; diagnosed with diabetes; unhoused; immigrant; smoking status; drug use status.

Municipality: Annual incidence of TB in municipality of treatment per 100 000 people (2015–2018 average); Mean number of healthcare workers in the municipality where they are receiving treatment (2018); Mean household income per capita in the municipality where they are receiving treatment (2010); Percent of population living in urban settings where they are receiving treatment (2010); Percent of population living in low-income settings where they are receiving treatment (2010; Percent of population living in favelas (2010). Further details on covariates are available in the Supplement.

Abbreviation: DOT, directly observed therapy.

^a^The model containing all individual and municipality covariates represents the primary analysis.

^b^Scenario 1 represents a DOT scale-up scenario where 50% of individuals currently not receiving DOT begin to receive DOT.

^c^Percentage point change refers to the absolute change in treatment successes. For example, a 2.3 percentage point change results in estimated treatment success of 81.7%: (79.4% baseline treatment success + 2.3% = 81.7%).

Several scenario analyses explored the effect of expanding DOT coverage. Under a scenario that assumed increases in DOT utilization to reduce non-DOT receipt by 50% in each municipality, individuals newly receiving DOT were estimated to have on average, under the model, a 26.0% (22.2, 29.8) reduction in the probability of unsuccessful treatment, equivalent to a 5.5 (4.7, 6.3) percentage point increase in success rates (Table 2). Averaged across the overall study population this implied a 7.1% (6.1, 8.2) reduction in the probability of unsuccessful treatment, and a 1.5 (1.3, 1.7) percentage point increase in the national success rate for TB treatment. Similar effects were seen in other scenario analyses (Supplementary Table 3.1).

### Sensitivity Analysis

We tested the robustness of estimated outcomes to a range of alternative analytic specifications, including changes to the regression model, approaches for dealing with missing data, inclusion criteria for the study population, and different exposure variables. Table 3 reports ORs for treatment success under these different models. When adjusting for all individual and municipality covariates, the lowest OR (1.11, 95% CI: 1.07, 1.14) was estimated under the MLM with state and municipality random effects, while the highest OR (2.52, 95% CI: 2.39, 2.67) was estimated when individual-level DOT receipt was used as the exposure variable. All ORs were positive and statistically significant (*P* < .05).

**Table 3.** Odds Ratio for Treatment Success Estimated Under a Range of Alternative Analytic Specifications

Model	Univariate Model (95% CI)	Multivariate Model, All Individual Covariates (95% CI)	Multivariate Model, All Individual And Municipality Covariates (95% CI)
Main analysis (n = 278 007)	1.76 (1.66, 1.86)	1.50 (1.42, 1.59)	1.46 (1.38, 1.55)
Sensitivity analyses: alternative regression model			
Multilevel model with municipality and state random effects (n = 278 007)	1.81 (1.67, 1.95)	1.13 (1.10, 1.16)	1.11 (1.07, 1.14)
Sensitivity analyses: alternative missing data approaches			
Missing DOT information coded as DOT (n = 278 007)	1.63 (1.52, 1.74)	1.60 (1.50, 1.71)	1.45 (1.36, 1.55)
Missing DOT information coded as non-DOT (n = 278 007)	1.94 (1.83, 2.07)	1.60 (1.51, 1.69)	1.48 (1.40, 1.58)
Missing outcome information coded as unsuccessful treatment (n = 278 007)	1.79 (1.70, 1.89)	1.60 (1.51, 1.69)	1.59 (1.51, 1.69)
Complete case analysis (n = 179 641)	1.84 (1.71, 1.97)	1.67 (1.56, 1.79)	1.15 (1.12, 1.18)
Sensitivity analyses: alternative study populations			
Excluding recurrent TB (n = 255 028)	1.77 (1.68, 1.88)	1.51 (1.43, 1.60)	1.49 (1.41, 1.58)
Primary care facilities only (n = 173 969)	1.65 (1.55, 1.75)	1.53 (1.44, 1.62)	1.55 (1.46, 1.66)
Smear-positive individuals only (n = 144 642)	1.78 (1.68, 1.89)	1.59 (1.50, 1.70)	1.59 (1.49, 1.70)
Sensitivity analyses: alternative exposure variable			
Individual-level DOT receipt (n = 278 007)	2.52 (2.38, 2.68)	2.50 (2.36, 2.65)	2.52 (2.39, 2.67)
Sensitivity analyses: alternative outcome variable (n = 278 007)			
Lost to Follow-up (n = 27 510)	0.44 (0.42, 0.48)	.48 (0.45, 0.52)	0.49 (0.46, 0.53)
Death (n = 19 147)	0.73 (0.67, 0.80)	1.04 (0.95, 1.14)	1.02 (0.93, 1.12)

Abbreviations: DOT, directly observed therapy; TB, tuberculosis.

When death and loss to follow-up were examined as separate binary outcomes adjusting for all individual and municipality covariates, higher municipal DOT coverage was associated with lower odds of loss to follow-up (OR: 0.49, 95% CI: .46, .53) and had no statistically significant relationship with death during treatment (OR 1.02, 95% CI: .93, 1.12) (Table 3).

### Spatial Autocorrelation

All 3 municipal indicators (DOT coverage, TB treatment success, and missingness of DOT) showed significant positive spatial autocorrelation, with Global Moran's I of 0.24, 0.10, and 0.20, respectively. LISA revealed significant clusters of high and low values (High–High and Low–Low), as well as spatial outliers, for all 3 municipal indicators. The spatial distribution and number of significant clusters varied by indicator. Moran scatterplots and maps of clustering for each variable are available in the Supplement.

## DISCUSSION

This study investigated the relationship between DOT and TB treatment outcomes, using a large dataset of demographic and clinical data collected under routine programmatic conditions in Brazil. In our main analysis, we found the odds of treatment success to be 46% higher for individuals receiving DOT compared to non-DOT, after adjusting for a wide range of potential confounding factors. In scenario analyses based on this result, we found that expanding DOT coverage under different scenarios could increase national treatment success rates by as much as 2 percentage points and reduce the overall percent of people experiencing unsuccessful treatment by up to ∼9%.

The results estimated in this analysis require careful interpretation. Firstly, they represent marginal improvements in treatment success rates averaged across the variation in DOT coverage observed in Brazil. They should not be interpreted as the benefit that could be achieved by transitioning all people to DOT treatment, or the expected benefit for any individual. The benefits of DOT will likely vary across individuals, and for some DOT will be inappropriate or infeasible. Instead, the results of this analysis should be interpreted as the possible outcome of placing a higher relative priority on DOT treatment and achieving coverage rates closer to those of higher-coverage municipalities, while still accounting for individual preferences and relevant clinical factors. Second, these findings were estimated from observational data, without random assignment. While DOT is recommended as part of TB care in Brazil, individuals may still decline DOT. Individual characteristics associated with better or worse treatment outcomes may impact the provider's decision to recommend DOT or the individual's decision to participate, such that DOT recipients may differ systematically from nonrecipients. It is possible that ORs comparing DOT and non-DOT recipients could reflect these baseline differences, instead of being a causal effect of DOT.

To address this issue, we used municipality-level variation in DOT coverage (instead of individual DOT receipt) to estimate outcomes in the main analysis, as there is expected to be minimal correlation between municipality-level DOT coverage and individual-level predictors of treatment success. Compared to the main analysis, the OR for treatment success (OR = 2.52) was substantially greater when estimated using individual DOT receipt, emphasizing the potential for confounding with this variable. This OR for individual DOT receipt is consistent with other observational studies investigating predictors of treatment success in this setting [12, 24, 25]. While our estimation strategy addresses 1 major source of confounding, it is possible that omitted municipality-level variables (affecting both treatment success and DOT coverage) could influence results. In particular, we lacked direct measures of TB program strength, such as quality of case management or contact tracing intensity, instead relying on socioeconomic and health-system proxies. These proxies may incompletely capture differences in TB program performance between municipalities, leaving room for residual municipal-level confounding.

The sensitivity analyses provide some evidence of how results changed under alternative assumptions, with the ORs of treatment success estimated from these different models ranging from 1.1 to 2.5, but in all cases were positive and statistically significant. When we considered loss to follow-up and death as separate outcomes, higher DOT coverage was strongly associated with reduced loss to follow-up but showed no clear effect on mortality, suggesting that DOT primarily acts through preventing treatment interruption, while mortality is influenced by additional factors that may be less modifiable by DOT.

While these analyses provide some evidence of the robustness of results, this study still provides weaker evidence than studies with random allocation of participants to treatment arms and should be interpreted with caution. In addition, our primary model relates municipality-level DOT coverage to individual outcomes, and the scenario analyses extrapolate individual-level benefits from these municipality-level associations. Consequently, the projected effects for “individuals newly receiving DOT” should be interpreted as model-based averages under hypothetical changes in municipal DOT coverage, rather than as direct estimates of the effect of DOT initiation in specific individuals.

A strength of this study is its external validity, reflecting the performance of DOT in real-world, routine programs, and the use of municipality-level DOT coverage as a contextual exposure linked to individual outcomes across almost all Brazilian municipalities. The fact that less than half of our sample received DOT reflects programmatic realities in Brazil and many other settings. In our scenario analyses we found expanded DOT coverage could have major benefits for individuals newly receiving DOT (potentially reducing risk of unsuccessful treatment by one-quarter), yet still produce a modest 1–2 percentage point increase in national treatment success rates. This suggests that greater DOT access would be valuable for the additional individuals who receive it, but is only a partial solution to low treatment success rates, and should be considered one component of a comprehensive approach to improve TB program effectiveness. However, even modest increases in treatment success could yield substantial benefits, given the elevated morbidity and mortality risks faced by individuals with unsuccessful treatment. In a study of long-term mortality among individuals treated for TB in Brazil, individuals discontinuing treatment before completion experienced all-cause mortality rates 68% greater than successfully treated individuals, over an average 5-year follow-up period [26].

Our analysis also revealed geographic clustering of DOT coverage, TB treatment success, and missingness of DOT information across Brazilian municipalities, reflecting variation in TB program effectiveness and reporting across geographic areas. These spatial patterns suggest interventions to scale-up DOT may be more effective if tailored to local needs, rather than being implemented uniformly nationwide. To enable greater DOT uptake, Brazil has implemented several recent policies that involve low-intensity, patient-centered interventions including the creation of community-based DOT groups and digital adherence technologies such as video DOT (VDOT) [11, 14]. These interventions aim to improve access and support adherence, offering patient-centered alternatives to traditional facility-based DOT, particularly for underserved populations. While randomized controlled trials in several countries have not shown clear improvements in treatment outcomes [27, 28], these technologies may be cost-effective compared to facility-based DOT, and could be beneficial if adapted to local contexts [29, 30]. Evidence on the effectiveness and cost-effectiveness of these innovations in Brazil remains limited, making it challenging to recommend rollout without further studies to establish the benefits, costs, and feasibility of both community-based and digital approaches.

This study had several limitations. First, the national disease registry does not record information on some potentially relevant variables, including treatment adherence. As such we were unable to evaluate the impact of DOT receipt on adherence rates, or explore how variation in adherence impacted treatment success. Second, receipt of DOT was reported as a binary variable in SINAN, such that we were unable to distinguish variation in the quality of DOT or DOT modality used. Third, ∼28% of individuals were missing data on DOT receipt, and 15% on treatment outcomes. We used established methods for imputing these missing values, and tested the robustness of results to alternative imputation approaches. When we excluded all individuals with missing data (complete case) this produced a lower effect size for DOT (OR = 1.15), suggesting systematic differences between those with complete and missing data. Fourth, we use a different definition of treatment success than the Brazil MOH, which categorizes individuals without a recorded treatment outcome as unsuccessfully treated [16]. Using this definition, the estimate of current treatment success rates fell to 68% and the OR increased (OR = 1.59). Fifth, our analysis did not explore effect modification. However, in sub-group analyses we found that the OR was higher when analyses were restricted to individuals without recurrent TB, individuals receiving treatment at a primary healthcare facility, and individuals with a positive SSM at diagnosis, pointing to potential variation in the incremental value of DOT. Last, our main analysis is based on municipality-level DOT coverage, and the scenario analyses rely on associations; they cannot fully capture how DOT would be targeted or experienced by individuals.

Overall, using evidence from routine practice, this study found a positive association between DOT and successful TB treatment outcomes in Brazil, alongside large geographic variation in DOT coverage and treatment success rates. While just under half of individuals receive DOT as part of their TB care, expanding coverage could improve outcomes. Efforts to increase DOT uptake should be considered as part of a comprehensive approach for improving TB program effectiveness, both in Brazil and globally. Future studies should assess the cost-effectiveness and feasibility of expanding DOT through VDOT and similar patient-centered strategies, to inform policy and investment decisions for TB care.

## Supplementary Material

ofag434_Supplementary_Data

## Supplementary Data

Supplementary materials are available at *Open Forum Infectious Diseases* online. Consisting of data provided by the authors to benefit the reader, the posted materials are not copyedited and are the sole responsibility of the authors, so questions or comments should be addressed to the corresponding author.
