# Supplementary material for: The Impact of Directly Observed Therapy on Successful Tuberculosis Treatment: A Statistical Analysis of National Notifications Data
Source: Open Forum Infect Dis. 2026 Jul 16;13(7):ofag434. doi: 10.1093/ofid/ofag434 (PMC13397121; doi:10.1093/ofid/ofag434)
Supplement: ofag434_Supplementary_Data [file ofag434_supplementary_data.docx]

**Supplementary information**

Table of Contents

[1. Information on Methodology for Generalized Estimating Equations 2](#_Toc231575690)

[Table 1.1. Odds ratio of treatment success estimated under different regression models, based on municipality-level variation in DOT coverage. 2](#_Toc231575691)

[2. Information on constructing municipality-level covariates from observed data 5](#_Toc231575692)

[3. Policy Scenario Outcomes for All Models 6](#_Toc231575693)

[Table 3.1. Results of additional scenario analyses for generalized estimating equation models 7](#_Toc231575694)

[4. Sensitivity Analysis Results and Imputation Methodology 8](#_Toc231575695)

[Table 4.1. Comparison of missingness in covariates used for multiple imputation by chained equations, individuals with missing DOT information versus the whole cohort. 10](#_Toc231575696)

[Table 4.2. Results of Sensitivity Analyses Evaluating Impact of Missingness of Receipt of DOT on Coverage Estimates and Odds Ratios, using Generalized Estimating Equations 11](#_Toc231575697)

[5. Multi-Level Model (MLM) using R Package glmer() 12](#_Toc231575698)

[5.1a. Univariate Model 12](#_Toc231575699)

[5.1b. Demographic Variables Only Model 13](#_Toc231575700)

[Table 5.1b: Variance Inflation Factor for Demographic Variables Model 13](#_Toc231575701)

[5.1c. Demographic and Selected Baseline Covariates 14](#_Toc231575702)

[Table 5.1c: Variance Inflation Factor for Individual Variables Model 14](#_Toc231575703)

[5.1d. All Individual-Level Variables Model 15](#_Toc231575704)

[Table 5.1d: Variance Inflation Factor for Individual Variables Model 15](#_Toc231575705)

[5.1e. All Municipality-Level Variables Model 16](#_Toc231575706)

[Table 5.1e: Variance Inflation Factor for Municipality-Level Variables Model 16](#_Toc231575707)

[5.1f. Complete multi-level model 17](#_Toc231575708)

[Table 5.1f: Variance Inflation Factor for Full Variables Model 18](#_Toc231575709)

[6. Spatial Autocorrelation Additional Results 19](#_Toc231575710)

[Global Moran’s I 19](#_Toc231575711)

[Local Indicators of Spatial Association (LISA) 23](#_Toc231575712)

[7. Parameter outputs for the adjusted generalized estimating equation model with all individual and municipal covariates 28](#_Toc231575713)

[Table 7.1. Parameter outputs for the adjusted generalized estimating equation model with all individual and municipal covariates 28](#_Toc231575714)

[8. Directed Acrylic Graph (DAG) 31](#_Toc231575715)

[9. Supplementary Material References 32](#_Toc231575716)

## 1. Information on Methodology for Generalized Estimating Equations

For all Generalized Estimating Equation (GEE) estimates, the geeglm() command was used from R package geepack. For all models, it was assumed that all pairs within a cluster (defined as municipality where treatment was received) have the same correlation. The variables used in each of the GEE models are defined below:

### Table 1.1. Odds ratio of treatment success estimated under different regression models, based on municipality-level variation in DOT coverage.

| **Value** | **Model 1**  **(Univariate)** | **Model 2**  **(Demographic Covariates)** | **Model 3 (Demographic and Selected Baseline Covariates)** | **Model 3**  **(All Individual Covariates)** | **Model 5**  **(Municipality Covariates)** | **Model 6**  **(All Covariates)*** |
| --- | --- | --- | --- | --- | --- | --- |
| Odds ratio | 1.76  (1.66, 1.86) | 1.70  (1.60, 1.80) | 1.52  (1.44, 1.61) | 1.50  (1.42, 1.59) | 1.69  (1.59, 1.79) | 1.46  (1.38, 1.55) |
|  | **Covariates included in the model** | | | | | |
| Individual demographics | – | **+** | **+** | **+** | – | **+** |
| Other individual covariates | – | – | **+** | **+** | – | **+** |
| Municipality covariates | – | – | - | – | **+** | **+** |

Odds ratio represents the odds ratio for treatment success given treatment via DOT, as compared to no DOT. *Primary analysis. Values in parentheses represent 95% confidence intervals.

“+” indicates covariates included in model.

“–” indicates covariates excluded from model.

Individual demographics included age group, year of tuberculosis diagnosis, sex, race, and education.

Other individual covariates include smear microscopy test result, culture test result, chest x-ray test result, type of TB case (recurrent or new case), HIV diagnosis, alcohol use disorder diagnosis, diabetes diagnosis, prison population, unhoused population, migrant population, smoking status, drug use status, and health system level of care for treatment. All individual covariates were recorded before treatment initiation. For model 3, included individual covariates are HIV diagnosis, prison population, and health system level of care for treatment.

Municipality covariates included annual incidence rate of TB cases in the municipality per 100,000 people, number of healthcare workers in the municipality, household income per capita in the municipality, percent urban population in the municipality, percent low-income households in the municipality, and percent of the municipality residing in favelas. Several municipality covariates were excluded from the final models due to high collinearity (Variance Inflation Factor greater than 10): number of hospital beds in the municipality, total health spending per municipality, total population, homicide rate per 100,000 people.

**Univariate**

No covariates except the independent variable, municipality-level DOT coverage.

**Demographic Only**

-Municipality-level DOT coverage

-Age group at TB diagnosis (years)

-Year of TB diagnosis

-Sex

-Race

-Education level

**Demographic And Selected Baseline Covariates**

-Municipality-level DOT coverage

-Age group at TB diagnosis (years)

-Year of TB diagnosis

-Sex

-Race

-Education level

-HIV status

-Prison population

-Health facility level for treatment

**All Individual Covariates**

-Municipality-level DOT coverage

-Age group at TB diagnosis (years)

-Year of TB diagnosis

-Sex

-Race

-Education level

-TB Case type

-Sputum Smear Microscopy result

-Culture result

-Chest X-ray result

-HIV status

-Diagnosed with alcohol use disorder

-Diagnosed with diabetes

-Prison population

-Unhoused

-Immigrant

-Smoking status

-Drug use status

-Health facility level for treatment

**Municipality Covariates Only**

-Municipality-level DOT coverage (2015-2018 aggregate)

-Annual incidence of TB in municipality of treatment per 100,000 people (2015-2018 average)

-Mean number of healthcare workers in the municipality where they are receiving treatment (2018)^1,2^

-Mean household income per capita in the municipality where they are receiving treatment (2010)^3^

-Percent of population living in urban settings where they are receiving treatment (2010)^4^

-Percent of population living in low-income settings where they are receiving treatment (2010)^5^

-Percent of population living in favelas (2010)^6^

**All Individual and Municipality Covariates**

-Municipality-level DOT coverage

-Age group at TB diagnosis (years)

-Year of TB diagnosis

-Sex

-Race

-Education level

-TB Case type

-Sputum Smear Microscopy result

-Culture result

-Chest X-ray result

-HIV status

-Diagnosed with alcohol use disorder

-Diagnosed with diabetes

-Prison population

-Unhoused

-Immigrant

-Smoking status

-Drug use status

-Health facility level for treatment

-Municipality-level DOT coverage (2015-2018 aggregate)

-Annual incidence of TB in municipality of treatment per 100,000 people (2015-2018 average)

-Mean number of healthcare workers in the municipality where they are receiving treatment (2018)^1,2^

-Mean household income per capita in the municipality where they are receiving treatment (2010)^3^

-Percent of population living in urban settings where they are receiving treatment (2010)^4^

-Percent of population living in low-income settings where they are receiving treatment (2010)^5^

-Percent of population living in favelas (2010)^6^

## 2. Information on constructing municipality-level covariates from observed data

For each municipality, TB notifications and treatment outcome successes were aggregated for 2015-2018. The percentage of individuals experiencing treatment success in each municipality was calculated by taking the observed number of treatment successes and dividing by the total number of individuals reporting either receipt or non-receipt of DOT, including those with imputed estimates. Aggregate municipality-level DOT coverage was estimated by taking the number of individuals reporting receipt of DOT and dividing it by the number of individuals reporting either receipt or non-receipt of DOT, including those with imputed estimates. Similarly, the percent missing DOT was estimated by counting the number of individuals in a municipality with “NA” for DOT receipt in SINAN before multiple imputation, and dividing by the total number of individuals in the municipality (inclusive of those with and without information on receipt of DOT).

To calculate annual incidence of tuberculosis per municipality, first the average number of TB notifications annually per municipality was estimated by taking the total number of SINAN-TB notifications in each municipality from January 1, 2015 to December 31, 2018, and dividing by four. Then, the average annual number of TB notifications was divided by the 2018 population estimate for each municipality^7^ and multiplied by 100,000 to estimate the incidence of TB per 100,000 people in each municipality.

## 3. Policy Scenario Outcomes for All Models

Calculations for Scenarios:

- Total change in treatment successes was calculated as: (Number of predicted treatment successes in the scenario – Number of predicted treatment successes in the baseline).
- The percent point change in treatment success is calculated as: (mean percent treatment successes in DOT scenario – mean percent treatment successes in baseline scenario). For example, for the univariate model in scenario 1, the mean percent of treatment successes in the DOT scenario was 81.7%, and the baseline mean percent of treatment successes was 79.4. So, the percent point change in treatment success is: 81.7 – 79.4 = 2.3
- For estimates of reductions in unsuccessful treatments, values are presented as a percent of reductions for all unsuccessful treatments, compared to Scenario 0, the baseline/standard of care.
- Changes to DOT coverage were made at the municipality level. Numbers newly receiving DOT estimated as: (national DOT coverage, scenario – national DOT coverage, baseline) * number of TB cases. This is an ecological approximation, as the model does not assign DOT at the individual level; thus, results for “newly receiving DOT” represent average effects for this estimated group rather than explicitly identified individuals.
- A parametric bootstrap was used to estimate uncertainty around model-predicted policy impacts, using the MASS package for parameter simulation in R. For each model, we identified the vector of regression coefficients and their variance-covariance matrix, and generated 1,000 replicates by randomly drawing coefficients from the implied multivariate normal distribution. For each replicate, we recalculated model-predicted outcomes under each scenario. The 95% confidence intervals were taken from the 1,000 draws, representing the 2.5^th^ and 97.5^th^ percentiles for each calculated value across iterations.

Across scenarios, the national DOT coverage increased to 72.7%, 63.8%, and 81.6% in scenarios 1, 2, and 3, respectively.

| **Outcome** | | **Univariate** | **Demographic Covariates Only** | **Demographic and Selected Baseline Covariates** | **All Individual Covariates** | **All Municipality Covariates** | **All Individual and Municipality Covariates^a^** |
| --- | --- | --- | --- | --- | --- | --- | --- |
|  | ***Scenario analysis: Increase DOT coverage by 20% in each municipality (Scenario 2)^b^*** | | | | | | |
| Total increase in treatment successes | | 4301  (3815, 4819) | 3992  (3564, 4444) | 3090  (2686, 3490) | 2899  (2496, 3293) | 4416  (3929, 4899) | 2810  (2420, 3226) |
| Percentage point^c^ increase in treatment success (total program) | | 1.6  (1.4, 1.7) | 1.4  (1.3, 1.6) | 1.1  (0.9, 1.3) | 1.0  (0.9, 1.2) | 1.6  (1.4, 1.8) | 1.0  (0.8, 1.2) |
| Percentage reduction in unsuccessful treatment (total program) | | 9.1%  (8.2, 10.1) | 7.1%  (6.4, 7.9) | 5.5%  (4.8, 6.2) | 5.2%  (4.4, 5.9) | 7.4%  (6.6, 8.2) | 4.8%  (4.0, 5.5) |
| Percentage point^c^ increase in treatment success, (individuals newly receiving DOT) | | 8.4  (7.4, 9.4) | 7.7  (6.9, 8.6) | 6.0  (5.2, 6.8) | 5.6  (4.8, 6.4) | 8.6  (7.6, 9.5) | 5.5  (4.7, 6.3) |
| Percentage reduction in unsuccessful treatments, (individuals newly receiving DOT) | | 49.1%  (44.1, 54.5) | 38.3%  (34.2, 42.6) | 29.8%  (25.8, 33.6) | 27.8%  (23.9, 31.6) | 40.1%  (35.8, 44.4) | 25.7%  (22.0, 29.5) |
|  | ***Scenario analysis: Increase DOT coverage by 20% in each municipality (Scenario 3)^d^*** | | | | | | |
| Total increase in treatment successes | | 8391  (7446, 9394) | 7891  (7052, 8780) | 6071  (5273, 6854) | 5772  (4969, 6558) | 8524  (7607, 9439) | 5522  (4764, 6332) |
| Percentage point^c^ increase in treatment success (total program) | | 3.0  (2.7, 3.4) | 2.8  (2.5, 3.2) | 2.2  (1.9, 2.5) | 2.1  (1.7, 2.4) | 3.1  (2.7, 3.4) | 2.0  (1.7, 2.3) |
| Percentage reduction in unsuccessful treatment (total program) | | 17.8%  (15.9, 19.7) | 14.0%  (12.6, 15.6) | 11.0%  (9.6, 12.4) | 10.3%  (8.8, 11.7) | 14.4%  (12.8, 15.9) | 9.4%  (8.0, 10.7) |
| Percentage point^c^ increase in treatment success, (individuals newly receiving DOT) | | 8.3  (7.3, 9.3) | 7.8  (7.0, 8.7) | 6.0  (5.2, 6.8) | 5.7  (4.9, 6.5) | 8.5  (7.5, 9.4) | 5.5  (4.7, 6.3) |
| Percentage reduction in unsuccessful treatments, (individuals newly receiving DOT) | | 48.9% (43.9, 54.2) | 38.7%  (34.5, 42.9) | 29.9%  (25.9, 33.7) | 28.3%  (24.3, 32.1) | 39.5%  (35.3, 43.7) | 25.8%  (22.1, 29.6) |

### Table 3.1. Results of additional scenario analyses for generalized estimating equation models

^a^ The model containing all individual and municipality covariates represents the primary analysis.

^b^ Scenario 2 represents a DOT scale-up scenario where DOT coverage increases by 20% in all municipalities (up to 100% coverage).

^c^ Percentage point change refers to the absolute change in treatment successes. For example, a 2.3 percentage point change results in estimated treatment success of 81.7%: (79.4% baseline treatment success + 2.3% = 81.7%).

^d^ Scenario 3 represents a DOT scale-up scenario where DOT coverage is increased to 80% for all municipalities with coverage below 80%. Municipalities with DOT coverage of 80% or more at baseline remain unchanged.

DOT: Directly Observed Therapy

## 4. Sensitivity Analysis Results and Imputation Methodology

For the sensitivity analyses around missingness in the DOT outcome, four scenarios were considered:

1. All individuals with missing DOT information received DOT
2. All individuals with missing DOT information **did not** receive DOT
3. All individuals with missing outcome information had unsuccessful treatment
4. Complete Case (i.e. excluding those with missing information on DOT or treatment outcomes)

For the first two scenarios, individuals with missing information on receipt of DOT (n = 79,234; 29% of all eligible individuals) who were otherwise eligible for inclusion in the study based on our inclusion/exclusion criteria were kept in the study population, and assigned to either receipt/no receipt of DOT. Municipality-level coverage was re-estimated for each municipality after updating estimates of receipt of DOT. The third scenario analysis took all individuals with missing information on treatment outcomes (n = 42,056; 15% of all eligible individuals) and assumed that they had unsuccessful treatment, with no change to the imputed estimate of DOT receipt for individuals. The complete case scenario dropped all individuals with missing DOT or missing outcome information (n = 179,641 in final dataset) and estimates of municipality-level DOT coverage were re-calculated after dropping. For all scenarios, generalized estimating equations (GEE; R package: geeglm) were used to estimate the odds ratio of an individual successfully completing TB treatment as a function of municipality-level DOT coverage.

Multiple Imputation Methodology

Multiple imputation by Chained Equations was performed in R, using the package “mice”. Ten iterations were estimated for each individual. For multiple imputation, receipt of DOT was estimated for all individuals with missing information on DOT receipt, based on the covariates:

-Municipality-level DOT coverage

-Age at diagnosis

-Year of diagnosis

-Sex

-Race

-Education level

-TB case type

-Sputum Smear Microscopy result

-Culture result

-Chest X-ray result

-Extra-pulmonary tuberculosis diagnosis

-HIV status

-Diagnosed with alcohol use disorder

-Diagnosed with diabetes

-Level of the health facility where they received treatment

-Prison population

-Unhoused

-Immigrant

-Smoking status

-Drug use status

-TB incidence per 100,000 people in the municipality where they are receiving treatment

-Mean number of healthcare workers in the municipality where they are receiving treatment

-Mean household income per capita in the municipality where they are receiving treatment

-Percent of population living in urban settings in the municipality where they are receiving treatment

-Percent of population living in low-income settings in the municipality where they are receiving treatment

-Percent of population living in a favela in the municipality

Comparison of cohort with missing DOT information versus those with DOT information

The observed treatment success among those with missing information on DOT receipt is 48.8% (n = 38,636), compared to 79.4% observed in the full SINAN cohort. MICE was used to estimate missing values for DOT, treatment outcome, and any missing covariates for each observation. Table 4.1 presents information on how much missingness there was in the dataset for each covariate used to estimate receipt of DOT and treatment outcomes, comparing the full cohort and the cohort consisting only of those with missing information on receipt of DOT.

### Table 4.1. Comparison of missingness in covariates used for multiple imputation by chained equations, individuals with missing DOT information versus the whole cohort.

| Variable Name | Percent Missing (All, including those missing DOT) | Percent Missing (Those Missing DOT only) |
| --- | --- | --- |
| Age at diagnosis | 0% | 0% |
| Year of diagnosis | 0% | 0% |
| Sex | 0% | 0% |
| Race | 0% | 0% |
| Education level | 0% | 0% |
| TB Case Type | 0% | 0% |
| Sputum Smear Microscopy | 0% | 0% |
| Culture | 0% | 0% |
| Chest X-Ray | 0% | 0% |
| Extra-Pulmonary TB | 0% | 0% |
| HIV status | 12.4% | 0% |
| Diagnosed with alcohol use disorder | 0% | 0% |
| Diagnosed with diabetes | 0% | 0% |
| Health facility level | 1.5% | 1.4% |
| Prison population | 0% | 0% |
| Unhoused | 0% | 0% |
| Immigrant | 0% | 0% |
| Smoking status | 0% | 0% |
| Drug use status | 0% | 0% |
| Incidence of TB in municipality | 0.1% | 0.2% |
| Number of healthcare workers | 5.0% | 5.3% |
| Household income | 5.0% | 5.3% |
| Percent urban | 0% | 0% |
| Percent low-income | 0% | 0% |
| Percent living in favelas | 0.1% | 0.2% |

### Table 4.2. Results of Sensitivity Analyses Evaluating Impact of Missingness of Receipt of DOT on Coverage Estimates and Odds Ratios, using Generalized Estimating Equations

| **Scenario** | **Number of Individuals Receiving DOT (% National Coverage)** | **Number of Individuals Reporting Treatment Success** | **Odds ratio (95% Confidence Interval)** | |
| --- | --- | --- | --- | --- |
|  |  |  | **Univariate Model** | **Full Multi-Variate Model** |
| Receipt of DOT estimated by multiple imputation (primary analysis)  (n = 278,007) | 125,901  (45.3%) | 220,776  (79.4%) | 1.76  (1.66, 1.86) | 1.46  (1.38, 1.55) |
| Individuals with Missing DOT Information Receive DOT  (n = 278,007) | 179,362  (64.5%) | 220,776  (79.4%) | 1.63  (1.52, 1.74) | 1.45  (1.36, 1.55) |
| Individuals with Missing DOT Information do not Receive DOT  (n = 278,007) | 100,128  (36.0%) | 220,776  (79.4%) | 1.94  (1.83, 2.07) | 1.48  (1.40, 1.58) |
| Individuals with Missing Outcome Information Have Unsuccessful Treatments  (n = 278,007) | 125,901  (45.3%) | 188,634  (67.9%) | 1.79  (1.70, 1.89) | 1.59  (1.51, 1.69) |
| Complete Case Analysis  (n = 179,641)* | 91,641  (51.0%) | 149,998  (83.5%) | 1.84  (1.71, 1.97) | 1.15  (1.12, 1.18) |

*The complete case analysis includes only those with no missing information for receipt of DOT or for treatment outcomes. There are no imputed estimates for either outcome in the complete case dataset. For the other sensitivity analyses, imputed values were maintained for estimations aside from those values that were intentionally altered for each analysis.

## 5. Multi-Level Model (MLM) using R Package glmer()

As a comparison/exploratory analysis, a multi-level logistic regression (R package: glmer) was used to estimate the probability of an individual successfully completing TB treatment based on municipality-level DOT coverage, including both univariate and multivariate regressions. The model was built across three levels: individual (first level), municipality (second level), and state (third level). The logistic regression was used to estimate the odds of an individual successfully completing TB treatment, including random intercepts at the municipality and state level. The analysis was first run as a univariate regression with municipality-level DOT coverage, then using several multivariate regressions with a range of individual-level and municipality-level characteristics.

### 5.1a. Univariate Model

Where *i* is an individual, *j* is a municipality, and *k* is a state:

**Level One**

logit(P(Y_ijk_ = 1)) = *_0jk_*

**Level Two**

*_0jk_* = $\beta$_00k_ + $\beta$_01_*coverage_dot_municipalities_jk_* + *µ*_0jk_

**Level Three**

$\beta$_00k_ = γ_000_ + *r*_00k_

*µ*_0jk_ ~ *Ν* (0, *σ*_π_^2^)

*r*_00k_ ~ *Ν* (0, *σ*_β_^2^)

### 5.1b. Demographic Variables Only Model

Where *i* is an individual, *j* is a municipality, and *k* is a state:

**Level One**

logit(P(Y_ijk_ = 1)) = *_0jk_* +*_1_age_group_dx_ijk_* *_k_* +*_2_dx_year_ijk_* +*_3_sex_ijk_* +*_4_race_ijk_* +*_5_education_ijk_*

**Level Two**

*_0jk_* = $\beta$_00k_ + $\beta$_01_*coverage_dot_municipalities_jk_* + *µ*_0jk_

**Level Three**

$\beta$_00k_ = γ_000_ + *r*_00k_

*µ*_0jk_ ~ *Ν* (0, *σ*_π_^2^)

*r*_00k_ ~ *Ν* (0, *σ*_β_^2^)

### Table 5.1b: Variance Inflation Factor for Demographic Variables Model

| **Variable Name** | **Variance Inflation Factor** |
| --- | --- |
| Proportion DOT Coverage | 1.03 |
| Age Category | 1.55 |
| Diagnosis Year | 1.01 |
| Sex | 1.07 |
| Race | 1.22 |
| Education | 1.65 |

### 5.1c. Demographic and Selected Baseline Covariates

Where *i* is an individual, *j* is a municipality, and *k* is a state:

**Level One**

logit(P(Y_ijk_ = 1)) = *_0jk_* +*_1_age_group_dx_ijk_* +*_2_dx_year_ijk_* +*_3_sex_ijk_* +*_4_race_ijk_* +*_5_education_ijk_* +*_6_hiv_ijk_* + *_7_prison_ijk_* + *_8_treatment_facility_level_ijk_*

**Level Two**

*_0jk_* = $\beta$_00k_ + $\beta$_01_*coverage_dot_municipalities_jk_* + *µ*_0jk_

**Level Three**

$\beta$_00k_ = γ_000_ + *r*_00k_

*µ*_0jk_ ~ *Ν* (0, *σ*_π_^2^)

*r*_00k_ ~ *Ν* (0, *σ*_β_^2^)

### Table 5.1c: Variance Inflation Factor for Individual Variables Model

| **Variable Name** | **Variance Inflation Factor** |
| --- | --- |
| Proportion DOT Coverage | 1.07 |
| Age Category | 1.92 |
| Diagnosis Year | 1.06 |
| Sex | 1.12 |
| Race | 1.51 |
| Education | 2.63 |
| HIV | 2.22 |
| Prison | 2.85 |
| Health Facility Treatment Level | 2.59 |

### 5.1d. All Individual-Level Variables Model

Where *i* is an individual, *j* is a municipality, and *k* is a state:

**Level One**

logit(P(Y_ijk_ = 1)) = *_0jk_* +*_1_age_group_dx_ijk_* +*_2_dx_year_ijk_* +*_3_sex_ijk_* +*_4_race_ijk_* +*_5_education_ijk_* +*_6_smear_dx_ijk_* +*_7_culture_dx_ijk_* +*_8_chest_xray_dx_ijk_* +*_9_extrapulmonary_tuberculosis_ijk_* +*_10_hiv_ijk_* + *_11_alcohol_use_disorder_ijk_* + *_12_diabetes_ijk_* + *_13_prison_ijk_* + *_14_homeless_ijk_* + *_15_immigrant_ijk_* + *_16_smoker_ijk_* + *_17_drug_user_ijk_* + *_18_treatment_facility_level_ijk_*

**Level Two**

*_0jk_* = $\beta$_00k_ + $\beta$_01_*coverage_dot_municipalities_jk_* + *µ*_0jk_

**Level Three**

$\beta$_00k_ = γ_000_ + *r*_00k_

*µ*_0jk_ ~ *Ν* (0, *σ*_π_^2^)

*r*_00k_ ~ *Ν* (0, *σ*_β_^2^)

### Table 5.1d: Variance Inflation Factor for Individual Variables Model

| **Variable Name** | **Variance Inflation Factor** |
| --- | --- |
| Proportion DOT Coverage | 1.09 |
| Age Category | 2.20 |
| Diagnosis Year | 1.04 |
| Sex | 1.10 |
| Race | 1.47 |
| Education | 1.87 |
| Smear | 1.51 |
| Culture | 1.44 |
| Chest X-Ray | 1.23 |
| HIV | 1.38 |
| Alcohol Disorder | 4.43 |
| Diabetes | 3.12 |
| Prison | 8.63 |
| Homeless | 9.26 |
| Immigrant | 4.81 |
| Smoker | 4.17 |
| Drug Use | 3.70 |
| Health Facility Treatment Level | 3.00 |

### 5.1e. All Municipality-Level Variables Model

Where *i* is an individual, *j* is a municipality, and *k* is a state:

**Level One**

logit(P(Y_ijk_ = 1)) = *_0jk_*

**Level Two**

*_0jk_* = $\beta$_00k_ + $\beta$_01_*coverage_dot_municipalities_jk_* + $\beta$*_02_total_obs_jk_* + $\beta$*_03_mean_workers_jk_* + $\beta$*_04_household_capita_jk_* + $\beta$*_05_percent_urban_jk_* + $\beta$*_06_percent_lowincome_jk_* + $\beta$*_07_percent_favelas_jk_* + *µ*_0jk_

**Level Three**

$\beta$_00k_ = γ_000_ + *r*_00k_

*µ*_0jk_ ~ *Ν* (0, *σ*_π_^2^)

*r*_00k_ ~ *Ν* (0, *σ*_β_^2^)

### Table 5.1e: Variance Inflation Factor for Municipality-Level Variables Model

| **Variable Name** | **Variance Inflation Factor** |
| --- | --- |
| Proportion DOT Coverage | 1.14 |
| Total TB Cases in Municipality, 2015-2018 | 7.63 |
| Mean number of healthcare workers, municipality | 7.37 |
| Mean household income per capita, municipality | 5.97 |
| Percent of population living in urban setting, municipality | 2.72 |
| Percent of population that is low-income, municipality | 4.99 |
| Percent of population living in a favela | 1.48 |

### 5.1f. Complete multi-level model

Where *i* is an individual, *j* is a municipality, and *k* is a state:

**Level One**

logit(P(Y_ijk_ = 1)) = *_0jk_* +*_1_age_group_dx_ijk_* +*_2_dx_year_ijk_* +*_3_sex_ijk_* +*_4_race_ijk_* +*_5_education_ijk_* +*_6_smear_dx_ijk_* +*_7_culture_dx_ijk_* +*_8_chest_xray_dx_ijk_* +*_10_hiv_ijk_* + *_11_alcohol_use_disorder_ijk_* + *_12_diabetes_ijk_* + *_13_prison_ijk_* + *_14_homeless_ijk_* + *_15_immigrant_ijk_* + *_16_smoker_ijk_* + *_17_drug_user_ijk_* + *_18_treatment_facility_level_ijk_*

**Level Two**

*_0jk_* = $\beta$_00k_ + $\beta$_01_*coverage_dot_municipalities_jk_* + $\beta$*_02_total_obs_tb_jk_* + $\beta$*_03_mean_workers_jk_* + $\beta$*_04_household_capita_jk_* + $\beta$*_05_percent_urban_jk_* + $\beta$*_06_percent_lowincome_jk_* + $\beta$*_07_percent_favelas_jk_* + *µ*_0jk_

**Level Three**

$\beta$_00k_ = γ_000_ + *r*_00k_

*µ*_0jk_ ~ *Ν* (0, *σ*_π_^2^)

*r*_00k_ ~ *Ν* (0, *σ*_β_^2^)

### Table 5.1f: Variance Inflation Factor for Full Variables Model

| **Variable Name** | **Variance Inflation Factor** |
| --- | --- |
| Proportion DOT Coverage | 1.22 |
| Age Category | 2.24 |
| Diagnosis Year | 1.04 |
| Sex | 1.10 |
| Race | 1.62 |
| Education | 1.96 |
| Smear | 1.61 |
| Culture | 1.58 |
| Chest X-Ray | 1.28 |
| HIV | 1.47 |
| Alcohol Disorder | 4.83 |
| Diabetes | 3.25 |
| Prison | 9.09 |
| Homeless | 9.21 |
| Immigrant | 4.73 |
| Smoker | 4.42 |
| Drug Use | 3.99 |
| Health Facility Treatment Level | 4.42 |
| TB Incidence per 100,000 peopl in Municipality, 2015-2018 | 8.26 |
| Mean number of healthcare workers, municipality | 7.98 |
| Mean household income per capita, municipality | 6.81 |
| Percent of population living in urban setting, municipality | 2.69 |
| Percent of population that is low-income, municipality | 5.55 |
| Percent of population living in a favela | 1.59 |

## 6. Spatial Autocorrelation Additional Results

All spatial autocorrelation was done at the municipality level using first-order Queen contiguity for spatial weighting. Of 4,787 municipalities considered, the number of neighbors ranged from 0 to 21. Municipalities had a mean of 5.13 neighbors, and a median of 5 neighbors.

### Global Moran’s I

Table 6.1 shows results of the Global Moran’s I statistic, for municipality-level indicators. P-values were estimated using 99,999 permutations. Figures 6.1-6.3 show the Global Moran scatterplots for municipality DOT coverage, percent successful TB treatments, and percent missing DOT information, respectively. In a Moran scatterplot, the x-axis represents each municipality’s standardized value for the outcome. The y-axis represents the spatially lagged (weighted mean) value of the outcome in neighboring municipalities. Municipalities without neighbors (ex: islands) were excluded from Global Moran’s I.

#### Table 6.1 Global Moran’s I statistics for municipality-level indicators

| Indicator | Moran’s I | z-value | Expected I | p-value |
| --- | --- | --- | --- | --- |
| DOT coverage | 0.24 | 26.0 | –0.0002 | <0.001 |
| Percent TB treatment success | 0.10 | 11.2 | –0.0002 | <0.001 |
| Percent Missing DOT information | 0.20 | 21.0 | –0.0002 | <0.001 |


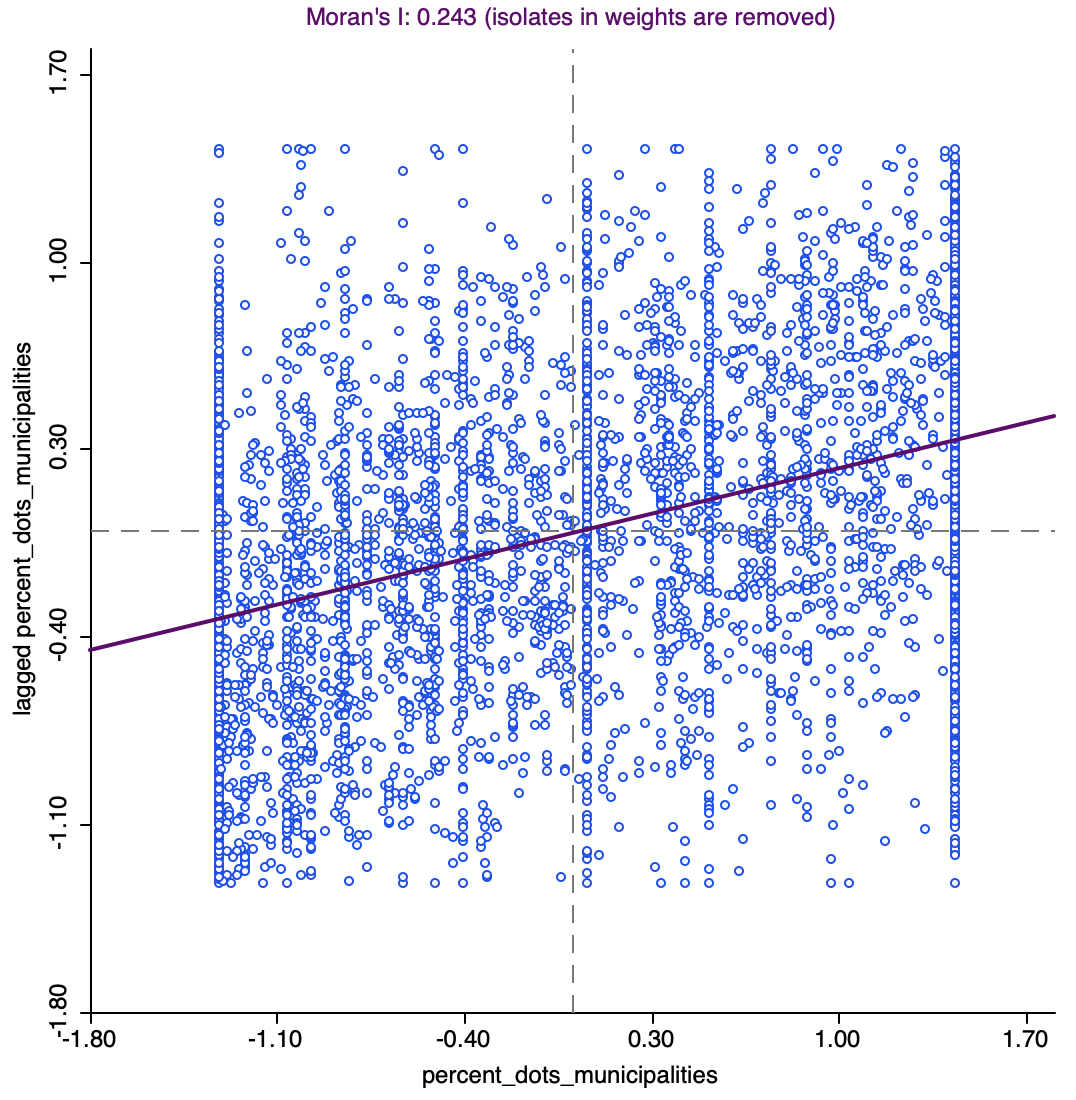


#### Figure 6.1. Global Moran’s I scatterplot for municipality-level DOT coverage.


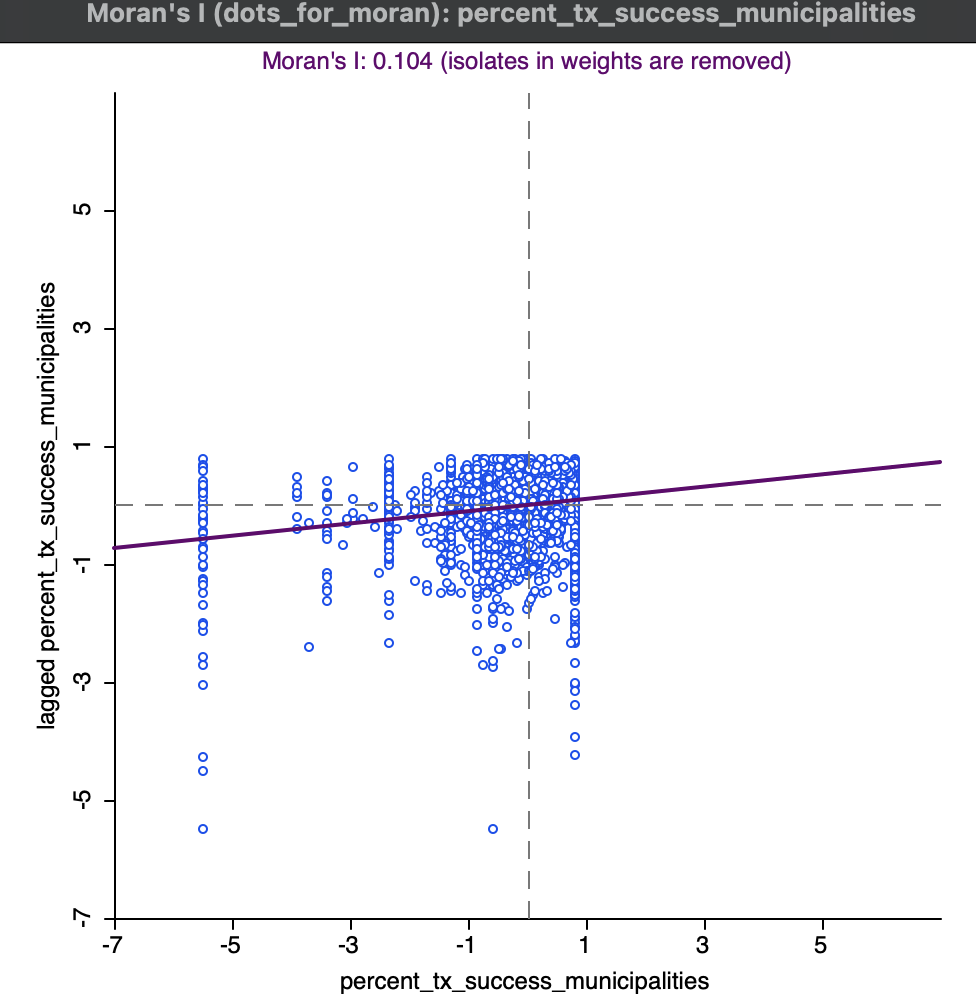


#### Figure 6.2. Global Moran’s I scatterplot for municipality-level proportion treatment successes.


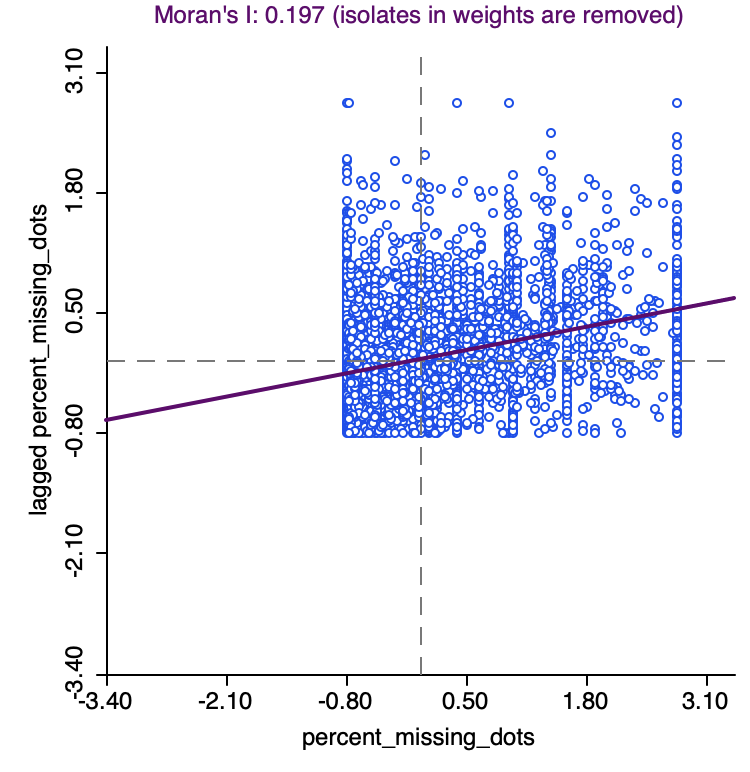


#### Figure 6.3. Global Moran’s I scatterplot for municipality-level DOT missingness (as a percent of all recorded people with tuberculosis in the municipality).

### Local Indicators of Spatial Association (LISA)

Municipality-level LISA cluster analyses were used to identify localized spatial autocorrelation for each municipality-level indicator using permutation-based p-values (99,999 permutations). Clustering can be defined as hot spots (High-High), cold spots (Low-Low), and spatial outliers (High-Low, Low-High). Overall, we saw notable differences in the geographic distribution and cluster counts across outcomes. Because each municipality represents a separate hypothesis test, we applied a false discovery rate (FDR) correction at α = 0.05 to account for multiple testing. This adjustment reduces the likelihood of false positives and ensures that only the strongest spatial clusters are retained.

For DOT coverage, 56 municipalities were classified as High-High clusters (i.e. high DOT coverage municipalities surrounded by high-coverage neighbors) and 80 as Low-Low clusters (low DOT coverage surrounded by low-coverage neighbors). Spatial outliers included 22 municipalities with high DOT coverage surrounded by low-coverage neighbors (High-Low), and 22 with low DOT coverage municipalities surrounded by high-coverage neighbors (Low-High). The remaining 4,605 municipalities were not significant for spatial autocorrelation. Figure 6.4a shows a map of localized spatial auto-correlation for DOT coverage.

For TB treatment success, no municipalities were identified as High-High clusters (municipalities with high levels of treatment success surrounded by high-success neighbors) and 12 as Low-Low clusters (municipalities with low levels of treatment success with low-success neighbors). There were 8 High-Low (high-success municipality with low-success neighbors) and 35 Low-High spatial outlier municipalities (low-success municipalities with high-success neighbors). Overall 4,730 municipalities were not significant for spatial autocorrelation for TB treatment success. Figure 6.4b shows a map of localized spatial auto-correlation for TB treatment success.

For missingness of DOT information, 34 municipalities were classified as High-High clusters (municipalities with a lot of missing information on DOT receipt, with high-missingness neighbors) and 18 as Low-Low clusters (municipalities with low levels of DOT missingness, with low-missingness neighbors). Looking at outliers, there were 27 High-Low (municipalities with high DOT missingness, with low-missingness neighbors) and 10 Low-High spatial outlier municipalities (low DOT missingness with high-missingness neighbors). The other 4,696 municipalities were not significant for spatial autocorrelation for DOT missingness. Figure 6.4c shows a map of localized spatial auto-correlation of DOT missingness.

1.
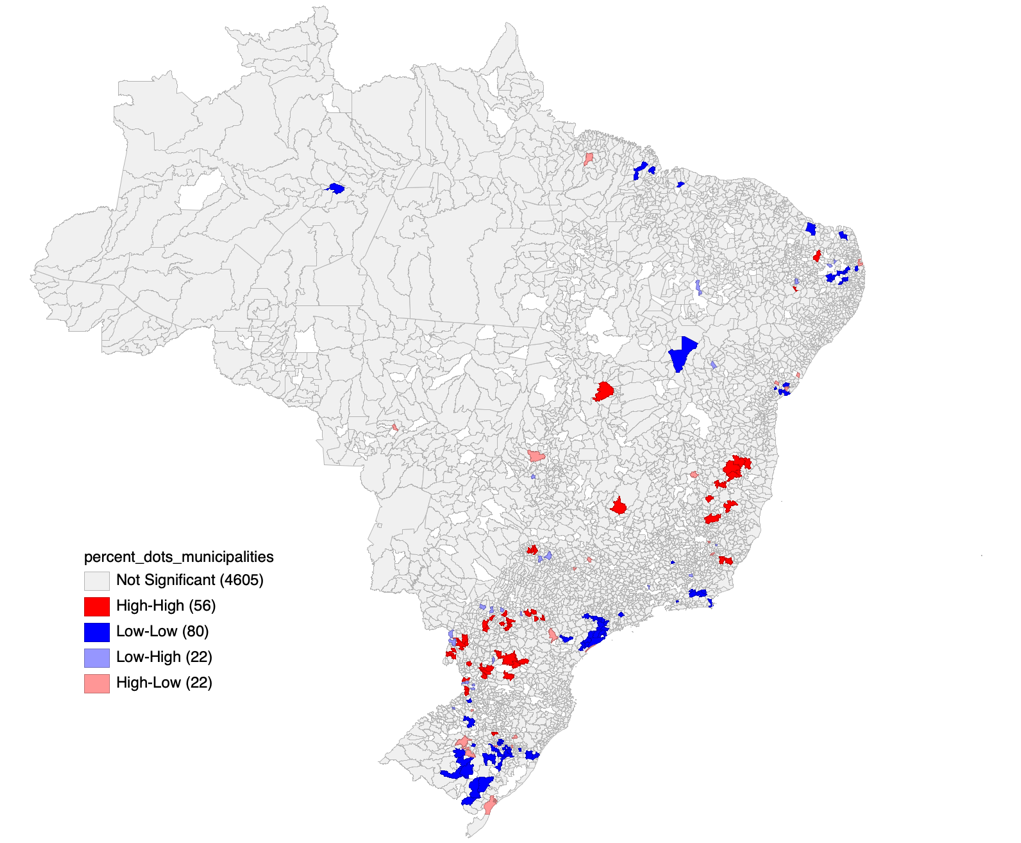

2.
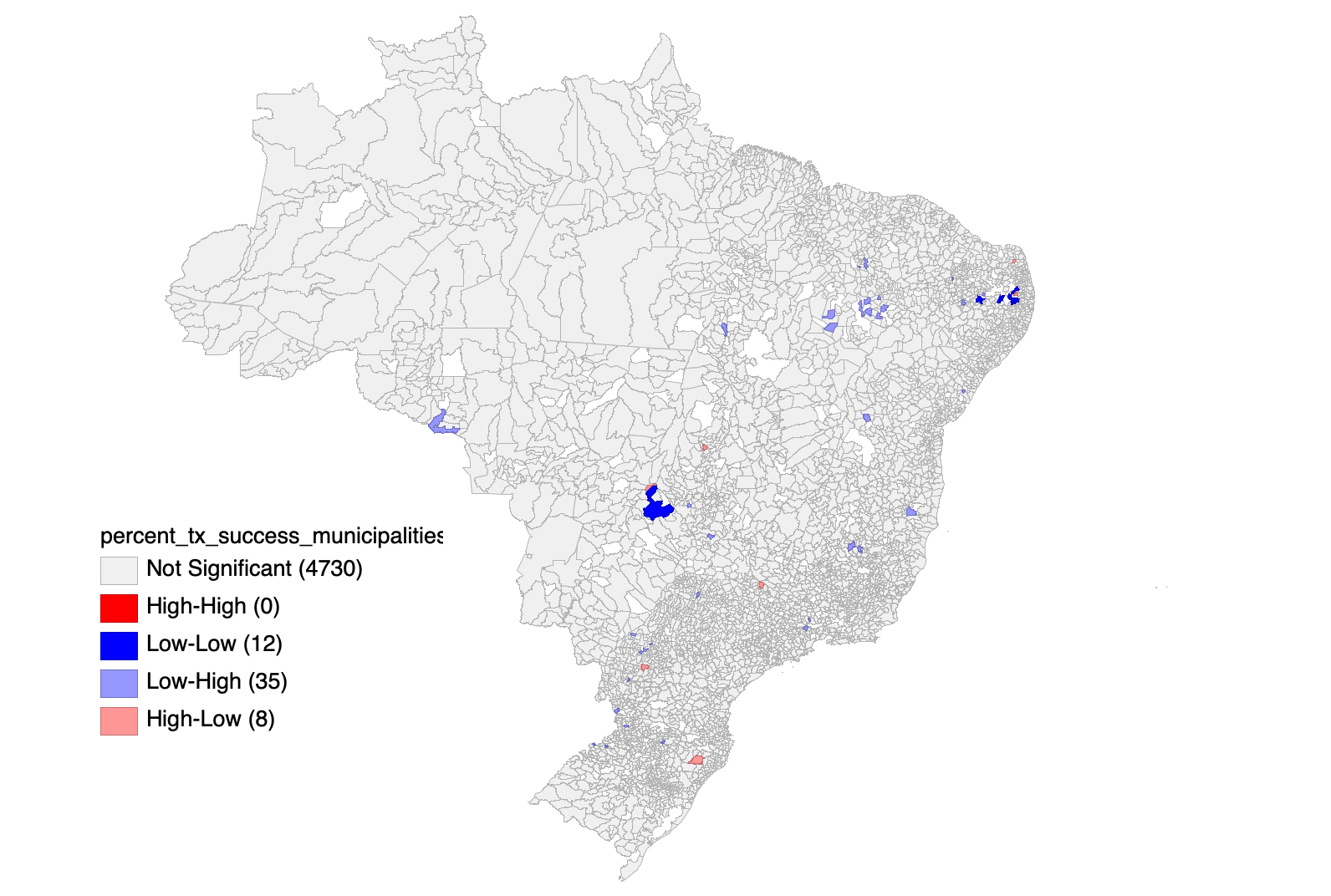

3.
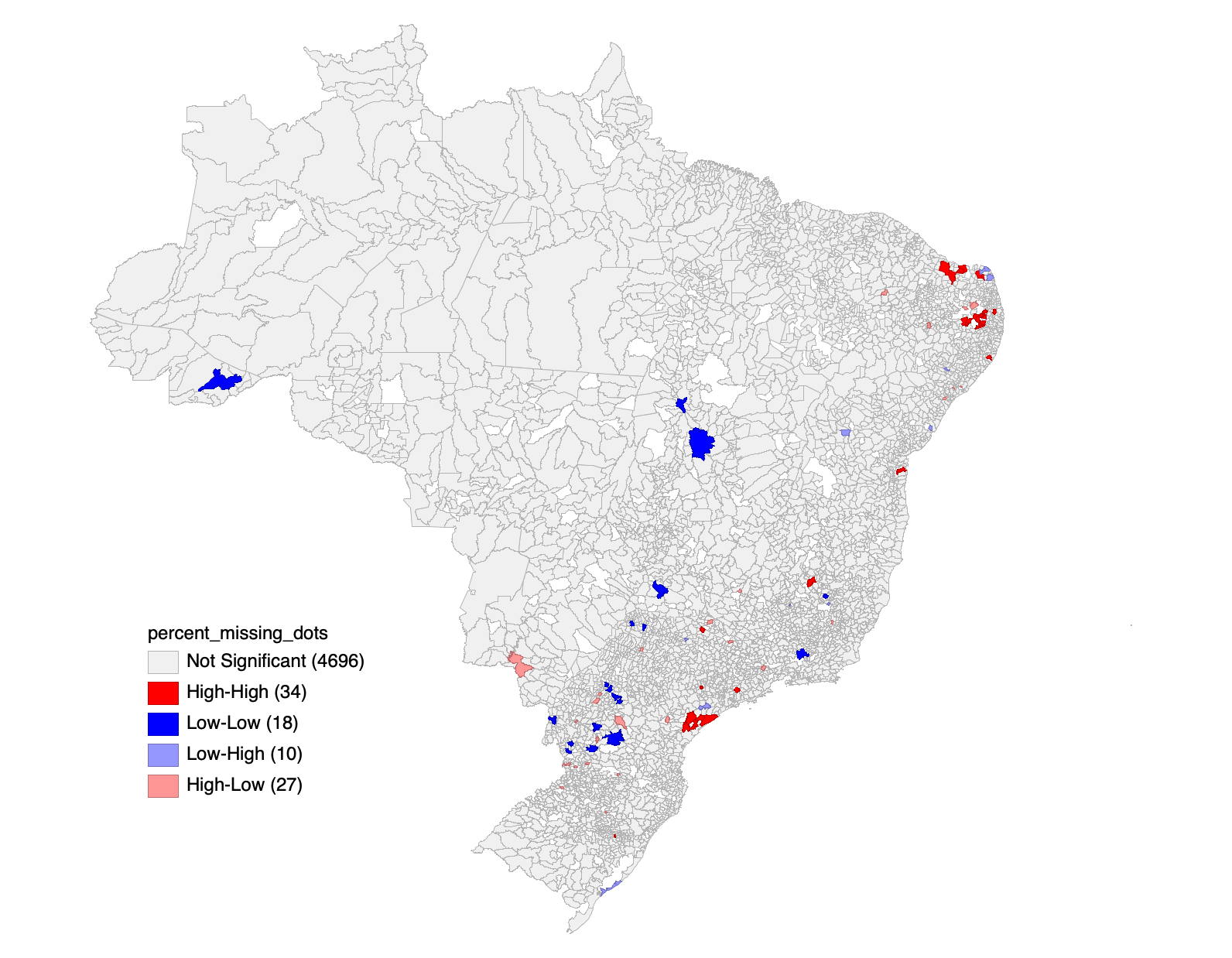


#### Figure 6.4. Maps representing municipality-level local indicators of spatial analysis (LISA) for (a) DOT coverage, (b) tuberculosis treatment successes, and (c) DOT missingness.

**Bivariate LISA**

DOT Coverage Vs Tuberculosis Treatment Success

Bivariate LISA analysis identified evidence of spatial co-clustering between DOT coverage and TB treatment success (Figure 6.5). No High-High clusters were detected (municipalities with both high DOT coverage and neighboring municipalities with high treatment success), but 15 Low-Low clusters were identified (municipalities with both low DOT coverage and neighbors with low treatment success). This pattern indicates spatial overlap of unfavorable program performance, suggesting that municipalities with low DOT coverage are often situated within broader regions experiencing similarly poor treatment outcomes.

Additionally, 6 municipalities were classified as High-Low outliers (high DOT coverage but surrounded by low treatment success), and 54 as Low-High outliers (low DOT coverage surrounded by high treatment success), reflecting areas where municipal DOT uptake diverges from treatment success in surrounding municipalities.


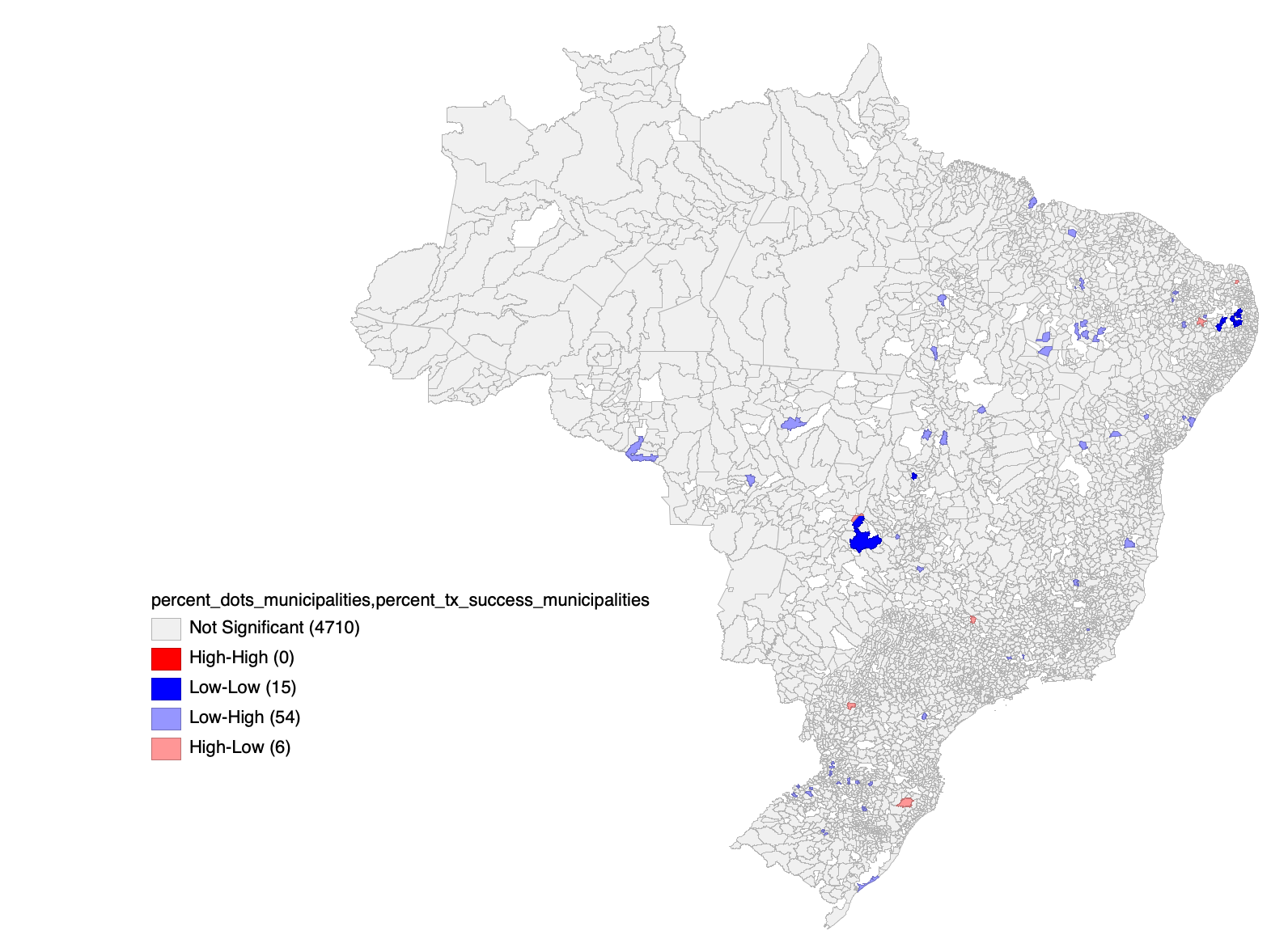


#### Figure 6.5. Map of bivariate LISA results for DOT coverage versus proportion of tuberculosis treatment successes, at the municipality level.

Missingness of Information on DOT Coverage Vs Tuberculosis Treatment Success

Bivariate LISA analysis identified evidence of spatial co-clustering between DOT missingness and TB treatment success (Figure 6.6). No High-High clusters were observed (municipalities with high DOT missingness and neighboring municipalities with high treatment success), but seven Low-Low clusters (municipalities with low DOT missingness and neighbors with low treatment success) were identified. Here, these Low‑Low clusters represent an unexpected pattern, as high DOT missingness is a poor outcome while high treatment success is desirable.

Additionally, 17 municipalities were classified as High-Low (a municipality with high DOT missingness, surrounded by municipalities with low treatment success), and 94 Low-High (low DOT missingness surrounded by high treatment success), where the performance of local and neighboring municipalities diverge from each other. As the covariates are inversely associated (high DOT missingness is a poor outcome, high treatment success is a favorable outcome), these outliers indicate areas where municipalities that excel in logging information on DOT receipt (low missingness) are spatially surrounded by areas that report high levels of treatment successes for TB treatment success, and vice versa. Depending on the nature of the missingness of the data, this could implicate an association between the quality of data reporting and TB care, with better reporting of DOT receipt spatially being correlated with successful treatment.


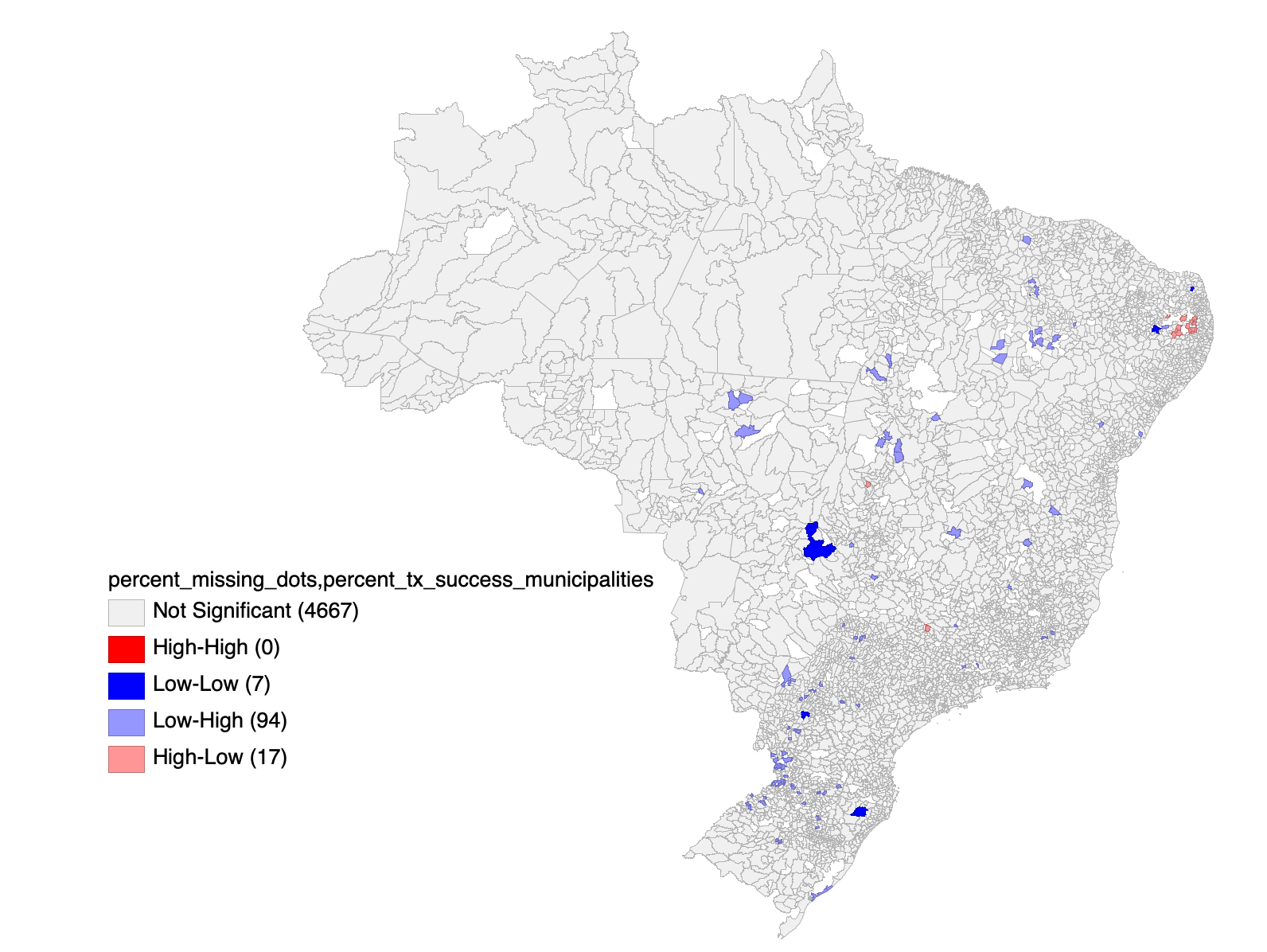


#### Figure 6.6. Map of bivariate LISA results for DOT missingness versus proportion of tuberculosis treatment successes, at the municipality level.

## 7. Parameter outputs for the adjusted generalized estimating equation model with all individual and municipal covariates

### Table 7.1. Parameter outputs for the adjusted generalized estimating equation model with all individual and municipal covariates

| **Variable** | **OR (95% CI)** |
| --- | --- |
| **Proportion Receive DOT, Municipality** | 1.46 (1.38, 1.55) |
| **Age Category** | |
| 0-4 | -- |
| 5-14 | 1.31 (1.13, 1.51) |
| 15-24 | 0.49 (0.44, 0.54) |
| 25-34 | 0.49 (0.44, 0.55) |
| 35-44 | 0.52 (0.47, 0.58) |
| 45-54 | 0.55 (0.49, 0.61) |
| 55-64 | 0.52 (0.47, 0.58) |
| 65-74 | 0.38 (0.34, 0.43) |
| 75-84 | 0.26 (0.23, 0.29) |
| 85+ | 0.18 (0.16, 0.21) |
| **Year of Diagnosis** | 0.91 (0.89, 0.92) |
| **Sex** | |
| Male | -- |
| Female | 1.25 (1.22, 1.28) |
| **Race** | |
| White | -- |
| Black | 0.79 (0.77, 0.82) |
| Asian | 0.96 (0.85, 1.08) |
| Mixed | 0.84 (0.82, 0.86) |
| Indigenous | 1.05 (0.92, 1.19) |
| Other | 0.98 (0.93, 1.03) |
| **Education** | |
| No Education | -- |
| Incomplete 1-4th grade | 1.11 (1.05, 1.17) |
| Complete 1-4th grade | 1.14 (1.08, 1.20) |
| Complete 5-8th grade | 1.40 (1.33, 1.48) |
| Complete high school | 1.92 (1.81, 2.05) |
| Higher education | 2.33 (2.18, 2.49) |
| Other | 1.02 (0.97, 1.08) |
| **Case Type** | |
| Incident TB | -- |
| Recurrent TB | 0.86 (0.83, 0.90) |
| **Sputum Smear Microscopy Result** | |
| Positive | -- |
| Negative | 0.89 (0.87, 0.92) |
| Not Performed | 0.85 (0.82, 0.88) |
| Not Applicable | 0.87 (0.82, 0.93) |
| **Culture Result** | |
| Positive | -- |
| Negative | 1.17 (1.12, 1.22) |
| In-Progress | 0.85 (0.81, 0.91) |
| Not Performed | 0.78 (0.75, 0.81) |
| Missing | 0.60 (0.51, 0.71) |
| **Health Unit Level** | |
| Primary Care | -- |
| Secondary Care | 0.75 (0.71, 0.78) |
| Tertiary Care | 0.29 (0.26, 0.33) |
| Other | 0.74 (0.66, 0.83) |
| **Chest X-Ray** | |
| Suspected TB | -- |
| Normal | 0.99 (0.95, 1.04) |
| Not Performed | 0.94 (0.92, 0.97) |
| **Type of Tuberculosis** | |
| Pulmonary TB | -- |
| Both Pulmonary and Extrapulmonary TB | 1.27 (1.23, 1.32) |
| **HIV Status** | |
| Positive | -- |
| Negative | 2.74 (2.63, 2.85) |
| Other | 1.35 (1.27, 1.44) |
| **Alcohol Use Disorder Status** | |
| Positive | -- |
| Negative | 1.42 (1.38, 1.47) |
| Other | 1.27 (1.16, 1.38) |
| **Diabetes Status** | |
| Positive | -- |
| Negative | 0.97 (0.93, 1.00) |
| Other | 1.06 (0.98, 1.16) |
| **Prison Population** | |
| Yes | -- |
| No | 0.46 (0.38, 0.56) |
| Other | 0.45 (0.36, 0.56) |
| **Homelessness** | |
| Yes | -- |
| No | 2.42 (2.27, 2.58) |
| Other | 2.27 (1.91, 2.69) |
| **Immigrant Status** | |
| Yes | -- |
| No | 1.16 (1.04, 1.30) |
| Other | 1.15 (0.99, 1.32) |
| **Smoker Status** | |
| Yes | -- |
| No | 1.19 (1.15, 1.22) |
| Other | 1.06 (0.99, 1.16) |
| **Drug Use Status** | |
| Yes | -- |
| No | 1.86 (1.80, 1.93) |
| Other | 1.57 (1.45, 1.70) |
| **TB Incidence, Municipality*** | 1.04 (1.01, 1.08) |
| **Mean Number of Workers, Municipality*** | 0.94 (0.91, 0.98) |
| **Mean Household Income per Capita, Municipality*** | 0.86 (0.82, 0.89) |
| **Percent Urban Population, Municipality*** | 0.93 (0.91, 0.95) |
| **Percent Low-Income Population, Municipality*** | 0.97 (0.94, 1.00) |
| **Percent of Population Living in Favelas, Municipality*** | 0.96 (0.94, 0.98) |

*Values with an asterisk indicate continuous variables that were standardized prior to modeling.

DOT: Directly Observed Therapy

TB: Tuberculosis

## Directed Acrylic Graph (DAG)


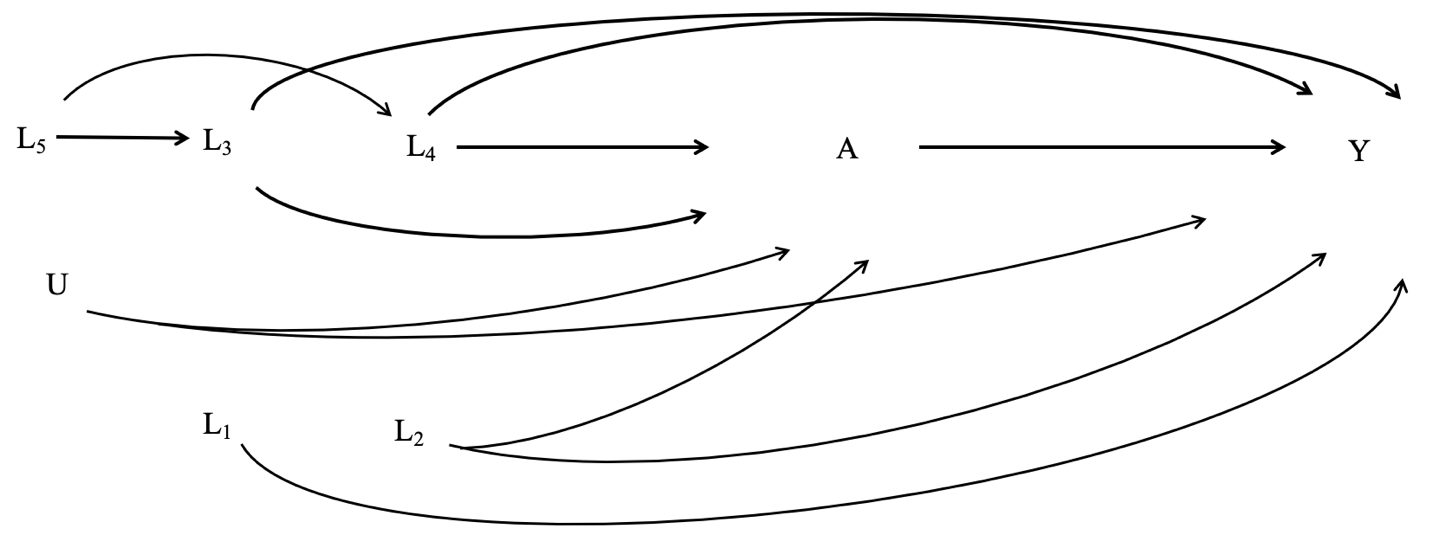


Figure 8.1 Directed Acylic Graph (DAG) to measure the effect of municipality-level DOT coverage on individual treatment success. The arrow from A to Y represents the causal effect of interest.

L_1_: Individual demographics (age group at TB diagnosis, year of TB diagnosis, sex, race, education level)

L_2_: Individual clinical attributes (TB case type (pulmonary, extra-pulmonary, both), lab test results (sputum smear, culture, chest X‑ray), comorbidities (HIV, diabetes)), and social-behavioral attributes (substance use, smoking status, unhoused, prison population, immigrant)

L_3_: Municipality-level socioeconomic factors (municipality TB incidence rate, percent of population in low-income settings, percent of population living in favelas, municipality-level household income per capita)

L_4_: Health system-level factors (health facility level for treatment, number of healthcare workers in the municipality)

L_5_: Urbanicity (percent of population in municipality living in urban settings)

U: Unmeasured confounders

A: Municipality-level DOT coverage (exposure)

Y: TB Treatment success (outcome)

DOT: Directly Observed Therapy

HIV: Human Immunodeficiency Virus

TB: Tuberculosis

## Municipality Characteristics

| **Variable** | **Low DOT coverage** | **Medium DOT coverage** | **High DOT coverage** |
| --- | --- | --- | --- |
| **DOT Coverage** | | | |
| Median (IQR), percent DOT coverage | 0 (0, 11.1) | 50 (34.5, 59.1) | 100 (82.4, 100) |
| Range (min, max), percent DOT coverage | 0, 25 | 25, 71 | 71, 100 |
| **Municipality Characteristics (Median, IQR)** | | | |
| Percent living in favelas* | 0.00 (0.00–0.00) | 0.00 (0.00–0.00) | 0.00 (0.00–0.00) |
| Percent low-income population | 41.4 (25.1–66.6) | 49.3 (26.4–68.0) | 43.2 (25.3–65.5) |
| Percent urban population | 68.0 (48.4–86.1) | 67.0 (48.8–83.9) | 66.4 (49.1–81.9) |
| Household income per capita | 475 (275–676) | 420 (262–627) | 462 (280–628) |
| Number of Healthcare Workers | 165.2 (77.0–391.0) | 170.1 (93.0–338.2) | 129.5 (72.3–257.0) |
| TB incidence per 100,000 | 14.6 (8.6–23.7) | 15.6 (10.3–24.2) | 12.7 (7.2–20.6) |
| Total TB notifications | 8.0 (2.0–27.0) | 9.0 (4.0–23.0) | 5.0 (2.0–15.0) |
| **State (Count, Percent of Municipalities within the State)** | | | |
| Acre | 0 (0%) | 2 (9.5%) | 19 (90.5%) |
| Alagoas | 30 (30.6%) | 44 (44.9%) | 24 (24.5%) |
| Amazonas | 21 (33.9%) | 26 (41.9%) | 15 (24.2%) |
| Amapá | 1 (7.1%) | 6 (42.9%) | 7 (50.0%) |
| Bahia | 164 (41.2%) | 145 (36.4%) | 89 (22.4%) |
| Ceará | 36 (19.8%) | 75 (41.2%) | 71 (39.0%) |
| Distrito Federal | 0 (0%) | 1 (100.0%) | 0 (0%) |
| Espírito Santo | 18 (23.4%) | 27 (35.1%) | 32 (41.6%) |
| Goiás | 50 (28.1%) | 58 (32.6%) | 70 (39.3%) |
| Maranhão | 90 (43.5%) | 76 (36.7%) | 41 (19.8%) |
| Minas Gerais | 166 (24.3%) | 224 (32.8%) | 292 (42.8%) |
| Mato Grosso do Sul | 13 (17.1%) | 39 (51.3%) | 24 (31.6%) |
| Mato Grosso | 32 (25.8%) | 57 (46.0%) | 35 (28.2%) |
| Pará | 63 (44.4%) | 58 (40.8%) | 21 (14.8%) |
| Paraíba | 72 (42.1%) | 65 (38.0%) | 34 (19.9%) |
| Pernambuco | 30 (16.4%) | 73 (39.9%) | 80 (43.7%) |
| Piauí | 50 (27.3%) | 70 (38.3%) | 63 (34.4%) |
| Paraná | 34 (9.8%) | 80 (23.1%) | 233 (67.1%) |
| Rio de Janeiro | 62 (68.1%) | 19 (20.9%) | 10 (11.0%) |
| Rio Grande do Norte | 60 (42.3%) | 35 (24.6%) | 47 (33.1%) |
| Rondônia | 27 (52.9%) | 12 (23.5%) | 12 (23.5%) |
| Roraima | 7 (58.3%) | 4 (33.3%) | 1 (8.3%) |
| Rio Grande do Sul | 229 (60.6%) | 83 (22.0%) | 66 (17.5%) |
| Santa Catarina | 76 (34.5%) | 69 (31.4%) | 75 (34.1%) |
| Sergipe | 12 (16.9%) | 22 (31.0%) | 37 (52.1%) |
| São Paulo | 230 (39.2%) | 202 (34.5%) | 154 (26.3%) |
| Tocantins | 22 (25.9%) | 21 (24.7%) | 42 (49.4%) |

* Median (IQR) values for the percent of the population living in favelas are 0.00 (0.00–0.00) in all DOT coverage tertiles because most municipalities have no recorded favela population. The mean (SD) percent of the population living in favelas was 1.54 (5.44) in the lowest DOT tertile (265 municipalities with any favela population), 1.26 (4.71) in the middle tertile (228 municipalities with any favela population), and 0.72 (3.68) in the highest tertile (156 municipalities with any favela population).

## Supplementary Material References

1. Ministério da Saúde, Cadastro Nacional dos Estabelecimentos de Saúde. Tabela: Team. 2025. Available at: <https://data-basis.org/dataset/354d6d98-bc09-4e22-a58a-e4eac3a5283c?table=58b8c805-9f83-42fc-a699-8b0abd34b109> (Accessed 17 June 2025).
2. Bigoni A, Malik AM, Tasca R, et al. Brazil’s health system functionality amidst of the Covdi-19 pandemic: an analysis of resilience. *Lancet Reg Health Am*. 2022; 10:100222.
3. O Instituto Brasileiro de Geografia e Estatística. Tabela 7610 – Rendimento nominal mensal domiciliar per capita dos domicílios particulares permanentes, por grandes regiões, unidades da federação e municípios. 2025. Available at: https://sidra.ibge.gov.br/tabela/7610 (Accessed 17 June 2025).
4. O Instituto Brasileiro de Geografia e Estatística. Tabela 1378 – População residente, por situação do domicílio (urbana/rural). 2025. Available at: https://sidra.ibge.gov.br/tabela/1378 (Accessed 17 June 2025).
5. O Instituto Brasileiro de Geografia e Estatística. Tabela 793 – População residente, por classes de rendimento nominal mensal domiciliar per capita, em salários mínimos. 2025. Available at: https://sidra.ibge.gov.br/tabela/793 (Accessed 17 June 2025).
6. O Instituto Brasileiro de Geografia e Estatística. Tabela 9900 – População residente em favelas e comunidades urbanas, total e indígena, por sexo e grupos de idade, segundo as Favelas e Comunidades Urbanas. 2025. Available at: <https://sidra.ibge.gov.br/tabela/9900#resultado> (Accessed 17 June 2025).
7. Ministério da Saúde, Departamento de Informática do SUS (DATASUS). População Residente – Estudo de Estimativas Populacionais por Município, Idade e Sexo 2000-2024 – Brasil. 2024. Available at: <http://tabnet.datasus.gov.br/cgi/deftohtm.exe?ibge/cnv/popsvs2024br.def> (Accessed 11 October 2025).
